# Abelson kinase’s intrinsically disordered region plays essential roles in protein function and protein stability

**DOI:** 10.1186/s12964-020-00703-w

**Published:** 2021-02-24

**Authors:** Edward M. Rogers, S. Colby Allred, Mark Peifer

**Affiliations:** 1 Department of Biology, University of North Carolina at Chapel Hill, Chapel Hill, NC 27599 USA; 2 Lineberger Comprehensive Cancer Center, University of North Carolina at Chapel Hill, Chapel Hill, NC 27599 USA

**Keywords:** Abl kinase, Intrinsically disordered region, Drosophila, Actin, Embryogenesis

## Abstract

**Background:**

The non-receptor tyrosine kinase Abelson (Abl) is a key player in oncogenesis, with kinase inhibitors serving as paradigms of targeted therapy. Abl also is a critical regulator of normal development, playing conserved roles in regulating cell behavior, brain development and morphogenesis. Drosophila offers a superb model for studying Abl’s normal function, because, unlike mammals, there is only a single fly Abl family member. In exploring the mechanism of action of multi-domain scaffolding proteins like Abl, one route is to define the roles of their individual domains. Research into Abl’s diverse roles in embryonic morphogenesis revealed many surprises. For instance, kinase activity, while important, is not crucial for all Abl activities, and the C-terminal F-actin binding domain plays a very modest role. This turned our attention to one of Abl’s least understood features—the long intrinsically-disordered region (IDR) linking Abl’s kinase and F-actin binding domains. The past decade revealed unexpected, important roles for IDRs in diverse cell functions, as sites of posttranslational modifications, mediating multivalent interactions and enabling assembly of biomolecular condensates via phase separation. Previous work deleting conserved regions in Abl’s IDR revealed an important role for a PXXP motif, but did not identify any other essential regions.

**Methods:**

Here we extend this analysis by deleting the entire IDR, and asking whether Abl∆IDR rescues the diverse roles of Abl in viability and embryonic morphogenesis in *Drosophila*.

**Results:**

This revealed that the IDR is essential for embryonic and adult viability, and for cell shape changes and cytoskeletal regulation during embryonic morphogenesis, and, most surprisingly, revealed a role in modulating protein stability.

**Conclusion:**

Our data provide new insights into the role of the IDR in an important signaling protein, the non-receptor kinase Abl, suggesting that it is essential for all aspects of protein function during embryogenesis, and revealing a role in protein stability. These data will stimulate new explorations of the mechanisms by which the IDR regulates Abl stability and function, both in Drosophila and also in mammals. They also will stimulate further interest in the broader roles IDRs play in diverse signaling proteins.

**Video Abstract**

## Background

Biomedical research has dual goals: to uncover mechanisms underlying normal cellular function and to apply this understanding to develop better treatments in human disease. Perhaps no story better illustrates this than the discovery more than 60 years ago of the “Philadelphia chromosome,” a translocation between chromosomes 9 and 22 present only in leukocytes from patients with chronic myelogenous leukemia. It provided the first molecular link between genetics and cancer, and ultimately led to the realization that Abelson kinase (Abl) is the initiating oncogene in many cases of chronic myelogenous and acute lymphoblastic leukemia [1]. These translocations fuse the *bcr* and *abl* genes, removing a myristoylation sequence at Abl’s N-terminus that inhibits kinase activation, rendering the kinase constitutively active. Drugs targeting Abl kinase activity like Gleevec (Imatinib) have emerged as paradigms of targeted therapy [2, 3], and spurred the development of similar inhibitors of other oncogenic kinases.

Of course, non-receptor tyrosine kinases like Src and Abl did not evolve to cause cancer. Both play key roles in signal transduction, embryonic development and tissue homeostasis. Abl family members regulate morphogenetic movements during embryogenesis in both mammals and *Drosophila*, and also play key roles in neural development, axon outgrowth, and synaptogenesis (reviewed in [4–7]. They act downstream of diverse receptors, including receptor tyrosine kinases, as well as the cell–matrix and cell–cell adhesion receptors, integrins and cadherins. Downstream, Abl family members activate cytoskeletal effectors to directly regulate cell behavior, though transcriptional effectors are also important.

**Fig. 1:**
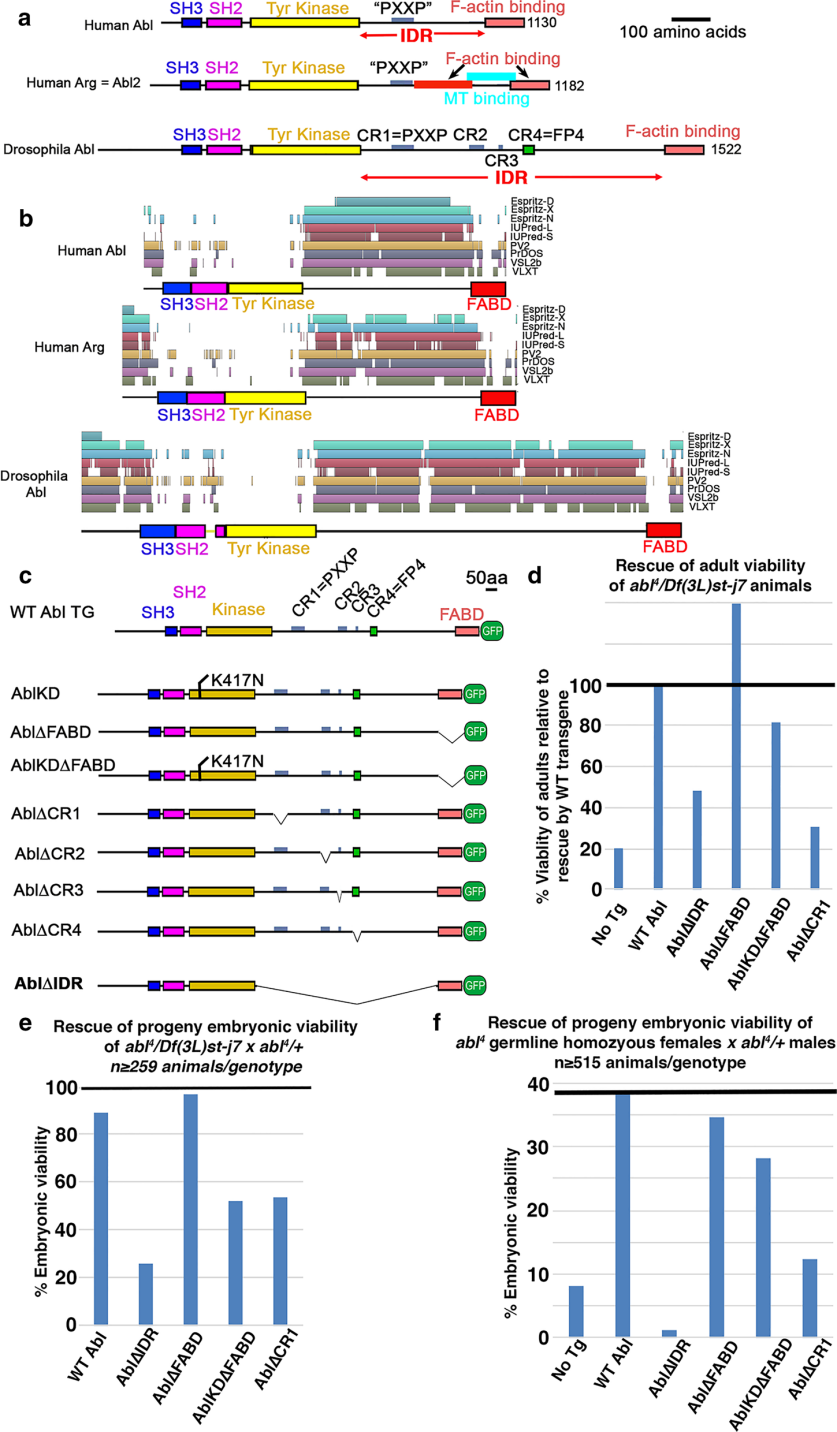
Generating Abl∆IDR and testing its ability to rescue adult and embryonic viability. **a** Diagram of human Abl and Arg and Drosophila Abl, showing conserved domains/motifs as well as motifs in the IDR that vary between family members. **b** All Abl family members share a region between the structured kinase and F-actin binding domains that is predicted to be disordered. Graphical report of unstructured regions from the D^2^P^2^ database for human Abl and Arg and Drosphila Abl. Nine different disorder predictions (depicted by the pastel-colored blocks) are stacked and aligned with the amino sequence for each protein (depicted in black) and the structured domains (depicted with colored blocks) within the polypeptide chain. **c** Illustration of the mutant Abl proteins we previously tested and our new Abl∆IDR mutant. It was designed to remove essentially the entire IDR, leaving only a few amino acids at each end to ensure we did not disrupt folding of the kinase domain or FABD. A 15 amino acid flexible linker was added in its place. **d** Assessment of the ability of Abl∆IDR to rescue the viability of *abl*^*4*^*/DfAbl* adults, normalized to rescue by our wildtype Abl transgene, and compared to rescue by some of our previously tested mutants. Line indicates degree of rescue relative to wildtype Abl transgene. Full data sets with statistical significance for D-F are in Table 1. **e** Assessment of the ability of Abl∆IDR to rescue embryonic viability of the progeny of *abl*^*4*^*/DfAbl* females mated to *abl*^*4*^*/*+ males, compared to rescue by some of our previously tested mutants. Line indicates 100% embryonic viability. **f** Assessment of the ability of Abl∆IDR to rescue embryonic viability of the progeny females with germlines homozygous for of *abl*^*4*^ mated to *abl*^*4*^*/*+ males, compared to rescue by some of our previously tested mutants. Line indicates degree of rescue by our wildtype Abl transgene

In order to understand Abl’s mechanism of action we need to define the roles of its different protein domains and regions. Abl’s structure facilitates the link between cell signaling and cytoskeletal regulation. All Abl family members share a highly conserved set of N-terminal domains with Src (Fig. 1a). These include a Src homology 2 (SH2) domain that binds specific peptides carrying a phosphorylated tyrosine and an SH3 domain that binds specific proline-rich peptides, both allowing interactions with upstream receptors and downstream effectors. These are immediately followed by the conserved tyrosine kinase domain [8]. However, unlike Src, Abl family members have long C-terminal extensions, with a C-terminal F-actin-binding domain (FABD) separated from the N-terminal module by a long linker that is less well conserved in primary sequence and that both prediction software and protease sensitivity assays suggest is intrinsically disordered (the intrinsically disordered region or IDR; [9]). We confirmed this using the D^2^P^2^ Database of Disordered Protein Predictions, which combines multiple computation approaches to predict disorder [10]—in human Abl and Arg and in *Drosophila* Abl the region between the kinase domain and the F-actin binding domain is predicted to be disordered (Fig. 1b). The region N-terminal to the SH3 domain, which varies in length among family members, is also predicted to be disordered (Fig. 1b). Different family members contain peptide motifs within the IDR that bind or are predicted to bind actin, microtubules, Ena/VASP family members, and SH3 domain containing proteins. The only peptide motif within the IDR clearly conserved between mammals and *Drosophila* is a PXXP motif that in mammals binds both SH2/SH3 adapters like Crk and Nck and the actin regulator Abl interacting protein (Abi) [11, 12].

Mammals have two Abl family members, Abl and Abl-related gene (Arg), with partially redundant functions in development and tissue homeostasis. *abl* single mutant mice die neonatally with thymic and splenic atrophy, T and B cell lymphopenia, osteoporosis, and cardiac defects [13–18]. Conditional knockout confirmed roles in T cells [19–21]. *arg* single mutant mice are viable with grossly normal brains, but exhibit multiple behavioral defects [22], likely linked to a reduced ability to maintain dendrites [23]. Arg mutants also exhibit subtle defects in muscle development [24]. In contrast, loss of both Abl and Arg leads to embryonic lethality at day 11, with a failure to complete neural tube closure. Conditional double knockout has revealed additional redundant roles in cerebellar [25], cerebral [26], and endothelial development and barrier function [27, 28].

*Drosophila* has a single Abl family member, simplifying analysis of its roles in development. In the 1980s Michael Hoffmann and his lab identified the first mutations in fly Abl, as part of a pioneering effort to define the normal roles of human oncogenes [29]. Others built on these efforts. Like its mammalian homologs, Abl plays important roles in embryonic and postembryonic neural development, acting downstream of diverse axon guidance receptors, including DCC/Frazzled, Robo, Plexin, Eph, and Notch (reviewed in [6]. Genetic and cell biological analyses also revealed Abl’s downstream effectors, the most prominent of which is Enabled (Ena), which binds the growing end of actin filaments and promotes their elongation. Abl negatively regulates Ena [30], through a mechanism that remains unclear. Trio, a GTP exchange factor (GEF) for the small GTPase Rac, is also an Abl effector.

Subsequent analysis of embryos lacking both maternal and zygotic Abl revealed additional roles outside of the nervous system. Abl regulates diverse events ranging from the actin-dependent cellularization process, to apical constriction of mesoderm precursors, cell intercalation during germband elongation, and collective cell migration during germband retraction, dorsal closure, and head involution [31–34]. In these events, regulation by and of the cadherin-based cell adhesion machinery plays a role, while Ena remains a critical downstream target [31–33].

In exploring the mechanism of action of multi-domain scaffolding proteins like Abl, one route is to define the roles of their individual domains. Abl kinase activity has been a focus of much attention, particularly after the success of Abl kinase inhibitors in the treatment of leukemia. However, the simplistic picture of Abl as a kinase acting solely by phosphorylating downstream proteins rapidly proved inaccurate. Kinase-dead Abl rescues defects in both adult viability and retinal development [35]. Analysis of its role in embryonic development suggests kinase activity is important for roles in both axon patterning and in morphogenesis, but a kinase-dead mutant retains significant residual function [36, 37]. More limited analysis implicated the SH2 domain in axon guidance [37]. The extended C-terminal region of Abl, including both the IDR and the C-terminal FABD, is essential for function, as *abl*^*1*^, which encodes a stable protein truncated soon after the kinase domain, behaves genetically as a null allele [29]. Similar results were seen in mice where a truncated protein has a null phenotype [13, 14]. The simplest explanation would have been that this reflected an essential function of the FABD. However, surprisingly Abl lacking the FABD fully rescues viability and fertility, though detailed analysis of axon patterning and synergistic effects with loss of kinase activity suggest the FABD does play a supporting role [9, 36, 37].

These data opened up potential roles for Abl’s IDR. IDRs like that in Abl are predicted to be unstructured in solution and often are rich in disorder-promoting amino acids (P, E, S, Q, and K). The past decade revealed unexpected and important roles for IDRs in diverse cell functions, including transcription, the DNA damage response, RNA metabolism, and cell signaling [38]. They are the preferred sites of many of the posttranslational modifications, including phosphorylation, acetylation, and ubiquitination, thus serving as the place cellular regulatory machinery can regulate protein function [39]. Alternative splicing of regions within the IDR can also alter their binding partners and thus function. As we found in Abl, they often contain short binding sites for other proteins and RNAs. These underlie their ability to mediate multivalent interactions, including those enabling assembly of “biomolecular condensates”. These condensates organize proteins and RNAs into non-membrane bound cellular compartments that perform the diverse functions outlined above [40, 41]. These multivalent interactions among their protein and RNA components can lead to “phase separation.” While not all proteins containing IDRs have been shown to form biomolecular condensates, intriguingly proteins containing SH2 and SH3 domains were among the first proteins shown to assemble by this mechanism [42], and Abl clearly can assemble into a large macromolecular complex (e.g. [12].

Abl’s IDR is poorly conserved in primary sequence, even between the two mammalian paralogs (Fig. 1a). Only a single peptide is conserved among fly and mammalian family members—the PXXP SH3-domain binding motif in the N-terminal quarter of the IDR. Other peptide motifs, including a predicted Ena binding site, are conserved over shorter phylogenetic distances; e.g., among insect Abl orthologs [36]. There are also functional motifs present only in single family members, including the microtubule and second actin interaction sites in mammalian Arg [43, 44]. Two groups assessed the functional roles of the *Drosophila* Abl IDR, taking different approaches. Our lab individually deleted short conserved regions of 12–56 amino acids (conserved regions 1 to 4 (CR1-CR4)), in the context of a GFP-tagged full length Abl construct driven by its endogenous promotor (Fig. 1c). We measured rescue of embryonic and adult viability, morphogenetic movements in the embryo, and axon outgrowth in the embryonic central nervous system [36]. Cheong and VanBerkum took a more comprehensive approach, deleting successively smaller fractions of the C-terminal IDR and FABD. They began by dividing it in half, then in quarters and finally focused in on two smaller regions, with smaller deletions and point mutations. They expressed their mutant proteins in the background of zygotic *abl* mutants using the GAL4-UAS system and assessed rescue of axon pathfinding [9]. Both approaches led to similarly surprising conclusions. Only the region containing the conserved PXXP motif plays a major role in Abl function. Surprisingly, however, this motif was extremely important, as its deletion reduced Abl function more than what was caused by loss of kinase activity or even loss of both kinase activity and the FABD. Subsequent analyses support the idea that this motif acts by interactions with the adapter protein Crk [45] and with the actin-regulatory WAVE complex [46]. The fine-grained dissections of the IDR by Cheong and VanBerkum suggest other regions of the IDR may have more subtle roles in axon guidance.

These data revealed that conserved sequences in the IDR play variable roles in Abl’s mechanism of action. However, these initial analyses did not fully probe the function of the IDR, as perhaps the simplest test of its function—completely deleting the IDR while leaving the FABD intact—was left out. Here, we directly test several mechanistic hypotheses about how Abl’s IDR contributes to Abl’s diverse functions during morphogenesis in vivo by generating a mutation that cleanly removes the IDR. This revealed essential roles for the IDR in embryonic and adult viability, in cell shape changes and cytoskeletal regulation during embryonic morphogenesis, and most surprisingly, in modulating protein stability.

## Materials and methods

### Transgenic fly lines

To create the AblΔIDR transgene, a pair of overlapping PCR products were generated with Phusion high fidelity DNA polymerase (NEB) using pUAS-Abl:GFP [33] as a template. pUAS-Abl:GFP contains 2 kb of 5′ upstream promoter from the endogenous *abl* gene as well as an in frame eGFP tag. The ΔIDR deletion was introduced by mutagenic DNA oligonucleotide primers in the overlapping section of the PCR products. In addition, a 15 amino acid flexible linker ((GGS)_5_) was added at the location of the deletion –the hydrophilic glycine and serine residues are unlikely to form secondary structures, reducing the likelihood that the linker will interfere with the folding and function of the adjacent kinase and FABD domains. The two overlapping PCR products were joined by PCR stitching and cloned into the XbaI/NotI fragment of pUASg-Abl:GFP to make pUASg-AblΔIDR:GFP. The primers used for mutagenesis were as follows:

AblΔIDR forward:

5′GGTGGATCCGGTGGATCAGGTGGATCCGGTGGTAGTGGTGGATCC**GCCACGCCTATTGCCAAACTGACCGAA**3′

AblΔIDR reverse:

5′GGATCCACCACTACCACCGGATCCACCTGATCCACCGGATCCACCG**GCTCCTCCGCCGGTGGCCACGCCCGA3**′

Italicized regions contain the code for the 15 aa flexible linker and the bold regions are complementary to the *abl* sequence. The resulting coding sequence spanning the deletion is: …TSGVATGGGAGGSGGSGGSGGSGGSATPIAKLTEP… The pUASg-AblΔIDR:GFP transgene was inserted via P-element transposition, and we were able obtain ten independent lines (three on the X Chromosome, two on the 2nd chromosome, and five on the 3rd chromosome). To make the targeted ΔIDR transgene, the insert was excised from pUASg-AblΔIDR:GFP with Xba1 and Not1 and ligated into pUASt-attP to make pUASt-attP-AblΔIDR:GFP. The targeted transgene was targeted to the left arm of the 2nd chromosome by phiC31 integrase-mediated transgenesis into PBac{yellow[+]-attP-3B}VK00037 (cytogenetic map position: 22A3; [47]. Injections of transgenic constructs were performed by BestGene Inc. and from these we obtained three independent lines.

### Fly stocks, viability and phenotypic analysis of *abl* mutants and statistical tests

All experiments were done at 25 °C unless noted. *y* w served as wildtype in our experiments. For assessing rescue of adult viability, we generated zygotic *abl* mutants by crossing *Df(3L)st-7 Ki/TM3 Sb* females to *transgene/transgene; abl*^*4*^*/TM3 Sb* males, and selecting for *Ki* and against *Sb* (*abl*^*4*^*/Df(3L)st-7 Ki*)*.* We set the fraction of progeny with this genotype seen when using the wildtype *abl* transgene (AblWT; 27%) as 100%, and other genotypes were normalized to this. Adult viabilities were compared by Fisher’s Exact test (GraphPad). For this, the number of viable mutant adult flies (# of *abl*^*4*^*/Df adults)* was compared to the estimated number of non-viable flies. The number of non-viable flies was estimated by subtracting the number of viable mutant adult flies from the expected number if they were fully viable (# of *abl*^*4*^*/TM3 plus Df/TM3 divided by 2*). We used two methods to generate embryos maternally and zygotically *abl* mutant (*ablM/Z*): (1) using the dominant female sterile method [48] to make *abl*^*4*^ clones in the female germline and (2) using a deficiency spanning the *abl* locus transheterozygous to *abl*^*4*^. To generate *abl* germline clones, w; *Tn[Abl]/Tn[Abl];FRT 79 D-F abl*^*4*^*/TM3* females were crossed with *hs::Flp;;FRT 79 D-F ovoD/TM3* males. 48–72 h old progeny were heat shocked for three hours at 37 °C and allowed to develop to adulthood. Virgin female progeny of the genotype *hs::FLP/*+*;Tn[Abl]/*+*; FRT 79 D-F abl4/FRT 79 D-F ovoD* were crossed with *w*; *Tn[Abl]/Tn[Abl];FRT 79 D-F abl*^*4*^*/TM3, twi-GAL4,UAS-EGFP* males*,* embryos collected from cups with apple juice/agar plates and yeast paste. To generate embryos and flies maternally and zygotically mutant for *abl* using a deficiency, we used *Df(3L) st-j7, Ki/TM6b* (Bloomington #5416, Deletes73A2-73B2). *w; Df(3L) st-j7, Ki/TM3, twi-GAL4,UAS-EGFP* females were crossed with w;*Tn[Abl]/Tn[Abl];FRT 79 D-F abl*^*4*^*/TM3* males. If the resulting w;*Tn[Abl]/*+*;FRT 79 D-F abl*^*4*^*/Df(3L) st-j7, Ki* females were viable, they were crossed to w;*Tn[Abl]/Tn[Abl];FRT 79 D-F abl*^*4*^*/TM3, twi-GAL4,UAS-EGFP* males and put into collection cups. and embryos collected. Assessment of embryonic lethality and preparation of embryonic cuticles were done as in Wieschaus and Nüsslein-Volhard (1986) [49]. Embryonic viabilities for both genetic approaches were compared by Fisher’s Exact test (GraphPad). Fisher’s Exact test (GraphPad) was also used to compare cuticle phenotypes of embryos expressing different Abl transgenes in an *abl*^*4*^*M/Z* background with either *abl*^*4*^*M/Z* mutant embryos or embryos expressing a wildtype Abl transgene in an *abl*^*4*^*M/Z* background. For each genotype, the number of cuticles falling into the two more severe classes (i.e. Dorsal closure failure, and Epidermal integrity defect) were grouped to a single defective category, and compared to the number of cuticles in the two less severe categories (wildtype and Strong defects in germband retraction).

### Embryo live imaging

Embryos from flies that homozygous for either the transgene encoding Abl WT or AblΔIDR were dechorionated in 50% bleach and mounted in halocarbon oil (series 700; Halocarbon Products, River Edge, NJ) between a gas-permeable membrane (Petriperm; Sartorius, Edgewood,NJ) and a glass coverslip and imaged in a Z-series of 1 μM slices on a Zeiss LSM-5 Pa confocal microscope (for Fig. 8h, i) or on a PerkinElmer UltraView spinning-disk confocal microscope (for Fig. 8j, k).

### Immunofluorescence

To examine embryos by immunofluorescence, flies were allowed to lay eggs on apple juice/agar plates with yeast paste for times calculated to obtain embryos at the right stages. Embryos were collected, dechorionated in 50% bleach, washed in 0.1% Triton-X, and fixed in 1:1 Heptane/3.7% Formaldehyde diluted in PBS for 20 min at room temperature. Embryos were then devitellinized by shaking in 1:1 heptane/methanol or when prepared for phalloidin staining, hand-devitellinized with a scalpel blade. Embryos were then blocked in Blocking Solution (PBS/0.1% Triton-X/1% Normal Goat Serum) for ≥ 30 min, incubated in primary antibody diluted in Blocking Solution overnight at 4 °C and washed 3X in Blocking Solution. Embryos were then incubated in secondary antibody in Blocking Solution for 2 h at room temperature and washed 3X in Blocking Solution. Embryos were mounted on glass slides in Aquapolymount (Polysciences, Inc). Primary and secondary antibodies were: (anti-Dcad, 1:100; anti-Enabled, 1:500; anti BP-102; 1:200; anti-Arm N27A1; 1:500 (all from the Developmental Studies Hybridoma Bank) and anti-mouse and anti-rat IgG Alexa Fluors 568 and 647, from Molecular Probes); some secondary antibodies were preabsorbed with fixed *y w* embryos. For F-actin staining TRITC labeled phalloidin (Sigma) was used at a dilution of 1:500 to 1:1000.

For S2 cells, resuspended cells were allowed to attach for 1 h onto a ConcanavalinA coated glass coverslip. Cells were then fixed 10 min in 10% formaldehyde HL3 buffer (70 mM NaCl; 5 mM KCl; 1.5 mM CaCl_2_-2H_2_O; 20 mM; MgCl_2_-6H_2_O; 10 mM NaHCO_3_; 5 mM trehalose; 115 mM sucrose; 5 mM HEPES; pH 7.2) followed by four 10 min washes in PBS with 0.1% Triton-X (PBST) and two brief washes with ddH_2_O. During the last PBST wash, TRITC labeled phalloidin was added to a dilution of 1:1000. A drop of Aquapolymount was added to the coverslips, and the coverslips were mounted on pedestals of dried nail polish on a glass slide, and sealed with nail polish. Imaging of embryos and S2 cells was done on a Zeiss LSM-5 Pa or Zeiss 710 scanning confocal microscopes. Images were processed using ZEN 2009 software. Photoshop CS6 (Adobe) was used to adjust input levels so that the signal spanned the entire output grayscale and to adjust brightness and contrast.

### Immunoblotting

Embryonic extracts for immunoblotting were prepared by resuspending embryos in an equal volume of 2X SDS-PAGE Sample buffer (100 mM Tris–Cl (pH 6.8);4% SDS; 0.2% bromophenol blue; 20% glycerol; 200 mM β-mercaptoethanol) and homogenizing with a pestle in a microfuge tube. To make S2 cell extracts 1 mL of resuspended S2 cells were spun down in a microfuge tube, the media was removed, and the pellet resuspended in an equal volume of 2X SDS PAGE Sample buffer. Samples were boiled for 5 min, spun to clear debris, and 10 μl of the resulting extract run on a 7.5% SDS-PAGE gel, and transferred to a nitrocellulose membrane. To detect the transgenic GFP-tagged Abl proteins we used anti-GFP (JL-8, 1:500 or 1:1000, Clontech). Anti-αTubulin (Sigma, 1:10,000) or anti-Pnut (Developmental studies Hybridoma Bank, 1:30) were used as loading controls. Detection was done using HRP-conjugated anti-mouse IgG secondary antibody (Pierce, 1:50,000), and the ECL plus substrate kit (Pierce).

### Quantification of Abl ΔIDR and Abl WT protein levels

Four immunoblots of embryo extracts from homozygous stocks of the targeted Abl WT and Abl ΔIDR transgenes were used to quantify relative levels of Abl WT and Abl ΔIDR proteins in the embryos. Scans of Western blot film exposures were opened and converted to grayscale images in Adobe Photoshop. The resulting image was opened in ImageJ as a JPEG and the pixels were inverted. Rectangular ROIs of the exact same dimensions, and just large enough to contain the thickest band were drawn around the Abl protein and loading control bands. An ROI was also drawn around an unexposed area of the film for background subtraction. The mean gray value (MGV) of the ROIs for Abl and loading control proteins, and background were determined. The background subtracted MGVs of the AblΔIDR and Abl WT bands were adjusted for any loading differences by dividing them by the MGVs of their background subtracted loading controls. The background and loading control adjusted AblΔIDR and Abl WT levels were expressed as a ratio of AblΔIDR/Abl WT, normalized to the level of Abl WT which was assigned a value of 1. To determine statistical significance an unpaired t-test was used (GraphPad).

### Expression of Abl proteins in S2 cells

To express Abl and Abl ΔIDR proteins in S2 cells, the Abl and Abl ΔIDR coding regions were cloned by Gateway Technology (Invitrogen) into pMT, a vector for metal inducible protein synthesis via the metallothionein promoter. To make pMT Abl::GFP and pMT AblΔIDR::GFP, Phusion Polymerase was used to amplify the Abl and Abl ΔIDR coding regions using pUASt-attP-Abl:GFP and pUASt-attP-AblΔIDR:GFP as a template with the following primers:

AblGFP gateway forward:

5′CACCATGGGGGCTCAGCAGGGCAA3′

AblGFP gateway reverse:

5′CCTGTTAAGCGCATTGGAGATCTGA3′

pMT Abl or pMT AblΔIDR::GFP DNAs were transfected into S2 cells grown in Sf-900 II SFM medium (Invitrogen) in the wells of 6 well plates (35 mm) using Effectene transfection reagent (Qiagen) according to the manufacturer instructions. Six hours after the transfection, CuSO_4_ was added to 500 mM to induce expression of the transgenes. Cells were allowed to induce for 24 h and were used for both Western Blots and Immunofluorescence microscopy. Transfection efficiency was estimated by counting GFP positive cells on a dozen 143 μm × 143 μm fields on slides for immunofluorescence and dividing by the total number of cell (for blot in Fig. 9b transfection efficiency: Abl WT = 65% (n = 205) and AblΔIDR = 57% (n = 109).

## Results

### Creating a mutant to test the role of the IDR in Abl function

Abl is a multidomain protein which uses both its kinase activity and its protein interaction domains to create a signaling hub, integrating upstream signals and activating downstream effectors. Our lab previously created a series of *abl* mutants to assess the role of kinase activity and other domains and motifs in *Drosophila* (Fig. 1c; [36]. The *Drosophila abl* gene encodes several splice variants that differ in the inclusion or exclusion of two exons: an 18 aa axon in the unstructured N-terminal region prior to the SH3 domain, and a 115 aa sequence in the C-terminal region of the IDR. The base construct for generating our mutants was a P-element transgene containing a C-terminally GFP-tagged wildtype *abl* cDNA driven by a 2 kb fragment of the 5′ upstream endogenous *abl* promoter (Tn Abl WT:GFP). The cDNA chosen encodes the isoform Abl-PG, which carries the N-terminal 18 aa alternate exon and lacks the 115 aa sequence in the IDR. This transgene can fully rescue *ablM/Z* null mutant embryos [33]. The C-terminal GFP tag does not impair its rescuing ability, and allows direct visualization of Abl localization in live embryos. The series of mutants included ones that deleted short conserved regions in the IDR (AblΔCR1-ΔCR4, Fig. 1c).

At that time we did not fully remove the IDR to assess its full set of roles. To test the mechanistic role of Abl’s IDR as a whole, here we created a similar transgene that essentially deletes the entire IDR—below we refer to this as Abl∆IDR (Fig. 1c; amino acids 679–1398 are deleted; see Methods for details). We added a 15 amino acid flexible linker ((GGS)_5_) in place of the IDR to reduce the likelihood of disrupting folding of the adjacent kinase domain and FABD [50]. We introduced this transgene into the *Drosophila* genome in two ways—by P element-based transformation (selecting an insertion on the second chromosome), and by site-specific integration on the left arm of the second chromosome (at 22A3).

**Fig. 2:**
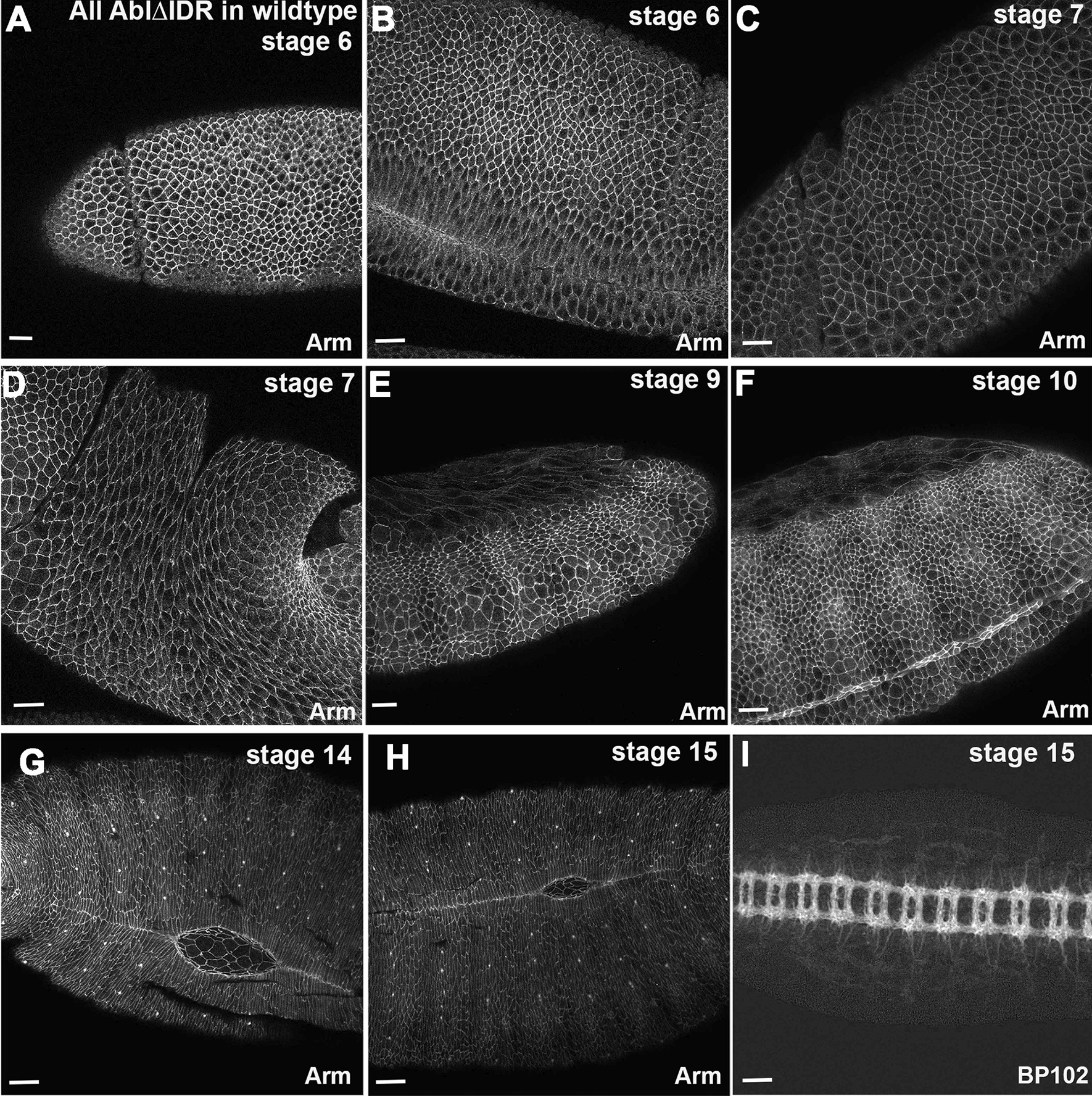
Abl∆IDR does not affect embryogenesis when expressed in a wildtype background. Embryos, anterior left, stages indicated, all expressing Abl∆IDR in a wildtype background. All are stained with antibodies to Armadillo (Arm), which outlines cell junctions, except I in which the central nervous system axons are visualized with the BP102 antibody—the channel showing the GFP signal is not shown. No apparent defects in morphogenesis were observed. Scale bars = 15 µm

We first assessed if expressing Abl∆IDR in the wildtype background had any dominant effect on viability or morphogenesis. We had no difficulty in creating Abl∆IDR transgenic lines, easily obtaining ten non-targeted and three targeted lines, and we have maintained two different lines of Abl∆IDR in the wildtype background for more than five years. This would have been prohibitively difficult if there had been any substantial lethality in a wildtype background. We also directly assessed embryonic lethality of flies expressing Abl∆IDR in the wildtype background. Neither of the two lines we tested had significant embryonic lethality—lethality was 3.7% and 9.6% for the two lines (n > 500 for each), both within the 3–10% range we observe for wildtype stocks. We also roughly assessed morphogenesis, by examining embryos expressing Abl∆IDR in the wildtype background. We did not observe any apparent defects in events in which Abl has a role [36], such as cellularization, ventral furrow invagination, germband retraction, dorsal closure or neural development (Fig. 2). We thus used this transgene to assess the roles played by Abl’s IDR in Abl’s regulation of embryonic morphogenesis.

### Abl’s IDR is essential for adult viability and embryonic morphogenesis

Because of its critical roles in embryonic morphogenesis and neuronal development, Abl is essential for both embryonic and adult viability [29, 31]. The ability to rescue viability thus offered an initial test for our Abl∆IDR mutant protein. Abl is maternally contributed and this maternal contribution is sufficient for embryonic development [31]. However, most *abl* null mutants die as pupae—the few that escape are functionally sterile and die soon after eclosing [29]. We thus tested the ability of Abl∆IDR to rescue adults that were heterozygous for the putative protein null allele *abl*^*4*^ [33] and a Deficiency, *Df(3L)st-7 Ki,* which removes the *abl* gene (*abl*^*4*^*/Df*), as we had done for our earlier mutants [36]. A targeted transgene encoding Abl∆IDR provided partial but incomplete rescue of adult viability. While unrescued *abl*^*4*^*/Df* adults had only 20% the viability of those rescued by our wildtype *abl* transgene, Abl∆IDR; *abl*^*4*^*/Df* adults eclosed at 48% the rate of those rescued by the wildtype transgene (Fig. 1d; Table 1; cross shown in Additional file 1: Fig S1). In contrast, Abl∆FABD fully rescued adult viability, and even a mutant lacking both kinase activity and the FABD provided substantial rescue [36]; 82% of the wildtype transgene). However, Abl∆CR1, lacking the PXXP motif in the IDR, did not provide substantial rescue (31% viability relative to the wildtype transgene; Fig. 1d; Table 1; [36]. These data suggest that the IDR is important for Abl’s wildtype mechanism of action.

**Table 1:** Comparison of AblΔIDR rescue of adult and embryonic viability with other Abl constructs

Construct	Residues changed or deleted	Percent rescue of adult viability in *abl*^*4*^*/Df(3L)st-j7* (normalized to WT)*	Probability that viability is higher than adults with no transgene	Probability that rescue is better or worse than that by Abl WT	Probability that rescue is better or worse than that by Abl ΔCR1	Percent rescue of progeny embryonic viabilty of *abl*^*4*^*/Df(3L)st-j7* X *abl*^*4*^*/*+**	Probability that rescue is worse than that by Abl WT	Probability that rescue is better or worse than that by Abl ΔCR1	Percent rescue of progeny embryonic viabilty of *abl*^*4*^* maternal germ line clone* X *abl4/*+***	Probability that rescue is worse than that of Abl WT	Probability that rescue is better or worse than that of *ablMZ*	Probability that rescue is better or worse than that by Abl ΔCR1
No Transgene	N/A	20.4 (n = 145)^#^	N/A	***p < 0.0001***	*p* = 0.4474	N/A	N/A	N/A	8.2% (n = 527)^#^	***p < 0.0001***	n/a	*p* = 0.0246
Abl WT	N/A	100% (n = 386)^#^	*p* < *0.0001*	N/A	*p* ≤ *0.0001*	89.4 (n = 901)^#^	N/A	*p* < *0.0001*	38.9% (n = 540)^#^	n/a	*p* < *0.0001*	*p* < *0.0001*
Abl∆IDR	679–1398	48.1% (n = 223)	*p* = *0.0075*	***p < 0.0001***	*p* = 0.3064	26.2% (n = 378)	***p < 0.0001***	***p < 0.0001***	1.1% (n = 569)	***p < 0.0001***	***p < 0.0001***	***p < 0.0001***
AblΔFABD	1418–1520	140% (n = 173)^#^	*p* < *0.0001*	*p* < *0.0001*	*p* < *0.0001*	97.1 (n = 362)^#^	*p* < *0.0001*	*p* < *0.0001*	34.6% (n = 515)^#^	*p* = 0.16	*p* < *0.0001*	*p* < *0.0001*
AblKDΔFABD	K417N 1418–1520	81.5% (n = 585)^#^	*p* < *0.0001*	***p = 0.0108***	*p* = *0.0029*	52.1 (n = 259)^#^	***p < 0.0001***	*p* = 0.7268	28.2 (n = 560)^#^	***p = 0.0002***	*p* < *0.0001*	*p* < *0.0001*
AblΔCR1	734–789	30.7% (n = 48)^#^	*p* = 0.4474	***p < 0.0001***	N/A	53.9 (n = 267)^#^	***p < 0.0001***	N/A	12.4% (n = 606)^#^	***p < 0.0001***	*p* = *0.02*	N/A

Comparison of AblΔIDR rescue of adult and embryonic viability in Abl null mutant background with other Abl constructs with statistical significance. A two-tailed Fisher's Exact Test was used to determine *p*-values of whether there was a statistically significant difference in extent of rescue between genotypes (see Methods)

^#^Data from Rogers et al. 2016 for comparison; n = number of adults/embryos scored; N/A = not applicable; italics = rescue better than indicated genotype; bold italics = rescue worse than indicated genotype

*Graphed in Fig. 1d

**Graphed in Fig. 1e

***Graphed in Fig. 1f

Unlike the *abl*^*4*^*/Df* escapers, Abl∆IDR; *abl*^*4*^*/Df* females lived long enough to mate and produce fertilized eggs. We thus asked whether Abl∆IDR rescued the lethality of embryos lacking both maternal and zygotic Abl, by crossing these females to males who were heterozygous for *abl*^*4*^*/*+ and carried the transgene. Abl∆IDR did not rescue the viability of maternal/zygotic mutants (which comprise 50% of the progeny), and, surprisingly, even 30% of embryos that inherited a paternal zygotic wildtype *abl* gene died before hatching and the rest (20%) died as first instar larvae (Fig. 1e, Table 1, cross shown in Additional file 1: Fig S1). In contrast, Abl∆FABD provided full rescue of embryonic viability (Fig. 1e; Table 1; [36].

**Fig. 3:**
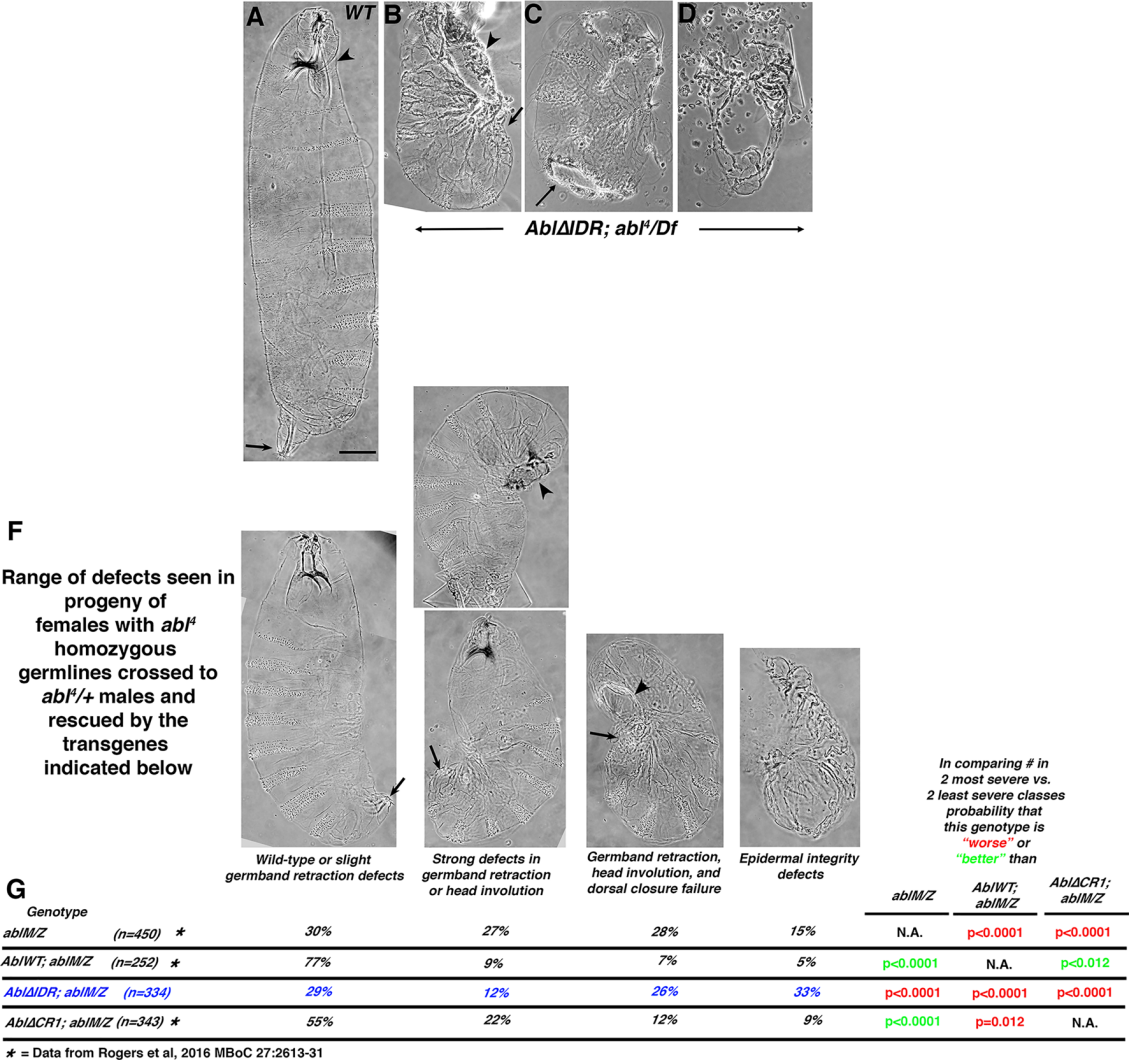
Abl∆IDR does not rescue embryonic morphogenesis. **a**–**f** Cuticle preparations. Anterior up. **a** Wildtype, ventral side right, revealing the segmental array of denticle belts and naked cuticle. Arrowhead: head involution was completed and there is a well-formed head skeleton. Arrow. Germband retraction was completed, positioning the spiracles at the posterior end. Scale bar = 50 µm. **b**–**d** Examples of cuticles from progeny of *Abl∆IDR; abl*^*4*^*/Df* mothers crossed to *abl*^*4*^*/*+ fathers. **b** Least severe phenotype. Head involution, dorsal closure (arrowhead) and germband retraction (arrow) failed. **c** Intermediate phenotype, with large hole in the ventral cuticle. **d** Severe phenotype. Only fragments of cuticle remain. **f** Range of cuticle defects seen in the progeny of females whose germlines are homozygous for *abl*^*4*^ crossed to *abl*^*4*^*/*+ fathers, carrying the transgenes indicated in G maternally and zygotically. Arrows and arrowheads as in **a**–**d**. Images in **a** and **f** are from Rogers et al., 2016, where we developed this cuticle scoring scheme. **g** *Abl∆IDR; ablM/Z* embryos have more frequent defects in epithelial integriy than either *Abl∆CR1; ablM/Z* embryos or even than unrescued *ablM/Z* embryos. Frequencies of each phenotype in the indicated genotypes. Statistical test used was Fisher’s Exact Test

Abl’s role in embryonic viability reflects its important roles in cell behavior in different tissues. To initially assess the role of Abl’s IDR in regulating morphogenetic movements, we examined cuticles of the embryonic progeny of Abl∆IDR; *abl*^*4*^*/Df* females. Examining cuticles allows us to assess cell fate choice, major morphogenetic movements like germband retraction, head involution, and dorsal closure, as well as epidermal integrity. To our surprise, the cuticle phenotype was quite severe, with all embryos exhibiting strong disruption of epidermal integrity, including those in which only fragments of cuticle were secreted (Fig. 3a vs. b–d). These morphogenetic phenotypes resemble the most severe phenotypes seen in *abl* maternal/zygotic mutant embryos [31, 36]. However, the limitations of this approach are that since unrescued *abl*^*4*^*/Df* mutant females are sterile, we could not compare embryonic morphogenesis of their progeny to those rescued by Abl∆IDR.

To circumvent this, we used the FLP/FRT/DFS approach [51] to generate females whose germlines are homozygous for *abl*^*4*^, either in the presence of one of our transgenes or in the absence of any transgene as a control. This approach allowed us to compare maternal/zygotic *abl* mutants (*ablM/Z),* who are homozygous for the null allele, with similar mutants that have one of our transgenes contributed both maternally and zygotically. We used *abl* transgenes inserted at the same chromosomal location via phiC integrase. *ablM/Z* mutants generated by the FLP/FRT/DFS approach are embryonic lethal [31], and there is only partial rescue of viability in the 50% of embryos that receive a wildtype *abl* gene paternally (9% overall embryonic viability (Fig. 1f; Table 1; cross shown in Additional file 1: Fig S1). Strikingly, *Abl∆IDR; ablM/Z* mutants had an even higher embryonic lethality (1% overall embryonic viability; probability that viability is lower than *ablM/Z p* < 0.0001; by Fisher’s Exact test; Table 1). In contrast, our GFP-tagged wildtype transgene provided strong rescue (39% viability [36]; we attribute the lack of full rescue to other mutations that have accumulated on the *abl*^*4*^ chromosome), as did the *Abl∆FABD* transgene (35% viability; [36]. Finally, these data revealed that Abl∆IDR rescues significantly less well than Abl∆CR1, suggesting the IDR contains additional important sequences (Fig. 1f; Table 1).

We next examined cuticles of Abl∆IDR; *ablM/Z* mutant embryos, assessing major morphogenetic movements like germband retraction, head involution, and dorsal closure, as well as epidermal integrity. *ablM/Z* mutants have multiple defects in these processes [31, 36]; Fig. 3f, g; Full data set in Additional file 2: Table S1), with most exhibiting strong defects in head involution and failure of full germband retraction. Many also fail in dorsal closure, and a small fraction (15%) have defects in epidermal integrity. Our transgene encoding wildtype Abl largely rescued these defects (Fig. 3f, g; [36]. Our previous analysis revealed that neither kinase activity nor the FABD is essential for rescuing these cuticle defects, while Abl∆CR1, which lacks the conserved PXXP motif within the IDR, largely rescued epidermal integrity but only partially rescued germband retraction and dorsal closure (Fig. 3f, g; [36]. In contrast, however, Abl∆IDR completely failed to rescue both defects seen in *ablM/Z* mutants, thus revealing it to be significantly more impaired in function than Abl∆CR1 (Fig. 3g). In fact, *Abl∆IDR; ablM/Z* mutants had even more severe cuticle defects than unrescued *ablM/Z* mutants, with the fraction of embryos with the more severe epidermal defects more than doubled, from 15 to 33% (Fig. 3f, g; probability that the cuticle defects are worse than *ablM/Z p* < 0.0001; by Fisher’s Exact test). This epidermal disruption phenotype was similar to what we observed in the progeny of *Abl∆IDR; abl*^*4*^*/Df* females (Fig. 3b–d). Taken together, the increased embryonic lethality and higher proportion of severe cuticle defects in these experiments and the unexpectedly severe cuticle phenotype seen in our initial *abl*^*4*^*/Df* experiments, suggested to our surprise that expressing Abl∆IDR not only fails to rescue loss of Abl, but also worsens some aspects of the *abl* null mutant phenotype in embryonic development.

### Abl∆IDR does not effectively rescue defects in cellularization or mesoderm invagination

Abl has diverse roles in embryonic development, ranging from regulating actin dynamics during syncytial development and cellularization to regulating apical constriction of mesodermal cells to regulating cell shape change and collective cell migration during germband retraction and dorsal closure. Our cuticle data suggested that Abl∆IDR was substantially impaired in Abl’s mechanism of action in morphogenesis. To test this mechanistic hypothesis, we used immunofluorescence and confocal microscopy to examine cell shape changes and cytoskeletal regulation during embryonic development, as we had done to assess the roles of kinase activity, the FABD, and the conserved motifs in the IDR [36].

**Fig. 4:**
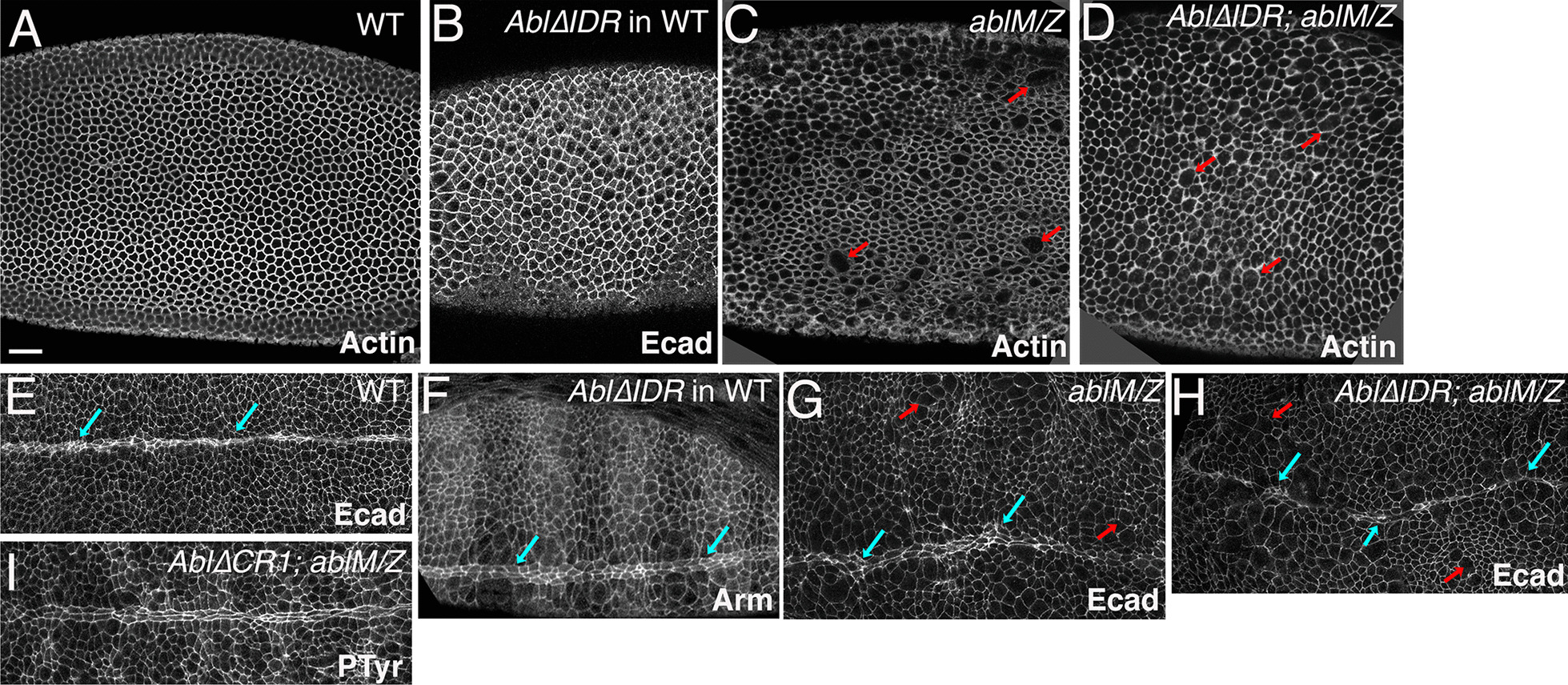
Abl∆IDR does not effectively rescue defects in cellularization or mesoderm invagination. Embryos, genotypes indicated, anterior left. **a**–**d** Cellularization, Phalloidin stained to reveal f-actin except B, is which Armadillo is visualized to reveal cell outlines. **a** Wildtype. Cellularization was completed normally, producing solely mononucleate cells. **b** Embryo expressing Abl∆IDR in a wildtype background. No presumed multinucleate cells are observed. **c** *ablM/Z* mutant*.* Defects in actin regulation during syncytial development and cellularization led to the formation of presumed multinucleate cells (red arrows). **d** *Abl∆IDR; ablM/Z* mutant. Abl∆IDR fails to rescue the defect in cellularization, and thus multiple presumed multinucleate cells are observed. **e**, **f** Stage 9 embryos, stained with antibodies to Ecad or Armadillo to visualize cell shapes. **e** In wildtype mesoderm invagination is completed normally leaving a straight and even midline (blue arrows). **f** Embryo expressing Abl∆IDR in a wildtype background. No defects in mesodermal invagination are apparent. **g** *ablM/Z* mutant*.* Defects in mesoderm invagination leave the ventral midline wavy and uneven (blue arrows). Also note the continued presence of presumed multinucleate cells (red arrows). **h** *Abl∆IDR; ablM/Z* mutant. Abl∆IDR fails to fully rescue the defect in mesoderm invagination, leaving a wavy midline (blue arrows). Multinucleate cells remain (red arrows). **i** *Abl∆CRI; ablM/Z* mutant. The mesoderm invagination phenotype is fully rescued. Scale bar = 15 µm

The first events of embryogenesis requiring Abl function are the characteristic dynamics of the actin cytoskeleton during the syncytial stages and cellularization. *ablM/Z* mutants have defects in both processes, and thus accumulate multinucleate cells at the end of cellularization [32]; Fig. 4a vs. c, red arrows), which persist throughout embryogenesis. Abl∆IDR did not appear to rescue these defects, as *Abl∆IDR; ablM/Z* mutants accumulated presumed multinucleate cells (Fig. 4a vs. d, red arrows). We quantified this later in embryonic development (Table 2; the full data set is in Additional file 3: Table S2)—during dorsal closure 3.9% of cells in *ablM/Z* embryos were presumably multinucleate (n = 5785 cells in five embryos), while 3.25% of cells in *Abl∆IDR; ablM/Z* embryos were presumably multinucleate (n = 7572 cells in five embryos). In contrast, expressing Abl∆IDR in a wildtype background had no apparent effects on the outcome of cellularization (Fig. 4b), nor did presumptive multinucleate cells accumulate in these embryos (0.39% presumptive multinucleate cells (3778 cells in two embryos) versus 0.23% in wildtype (n = 3899 cells in two embryos; Table 2, Additional file 3: Table S2). Abl is also required for the first event of gastrulation, in which cells along the ventral midline apically constrict in a coordinated way and invaginate as a tube [33]. The invaginating cells then go on to become mesoderm, while the ectodermal cells close the gap and form a straight midline (Fig. 4e). Expressing Abl∆IDR in a wildtype background had no apparent effects on this process (Fig. 4f). In *ablM/Z* mutants, apical constriction is poorly coordinated, leaving some mesodermal cells on the surface. Ectodermal cells eventually close the gap, but the resulting midline is not straight (Fig. 4e vs. g, blue arrows). Once again, Abl∆IDR did not fully rescue these defects (Fig. 4h; failure to rescue in 2/2 embryos examined at this stage). This latter phenotype is interesting as Abl∆CR1 fully rescues mesoderm invagination [36].

**Table 2:** Counts of presumptive multinucleate cells in AblΔIDR embryos

Genotype	Stage	Number of embryos scored	% presumptive multinuclear cells
*Abl*Δ*IDR; Abl M/Z*	Dorsal closure	5	3.45
*Abl M/Z*	Dorsal closure	5	3.90
*Abl*Δ*IDR*	Dorsal closure	2	0.39
*Wildtype*	Dorsal closure	2	0.22
*Abl*Δ*IDR; Abl M/Z*	Cellularization	2	4.02
*Abl M/Z*	Cellularization	2	10.02

Mean percentage of cells scored as multinucleate in embryos in the indicated stage of the indicated genotypes. For raw data used to generate this table see Additional file 3: Table S2. Examples of cells scored as multinucleate during dorsal closure are indicated in Fig. 6 by green asterixis. Examples of cells scored as multinucleate during cellularization are indicated in Fig. 4 by red arrows

### Abl’s IDR is essential for its roles in germband retraction and dorsal closure

**Fig. 5:**
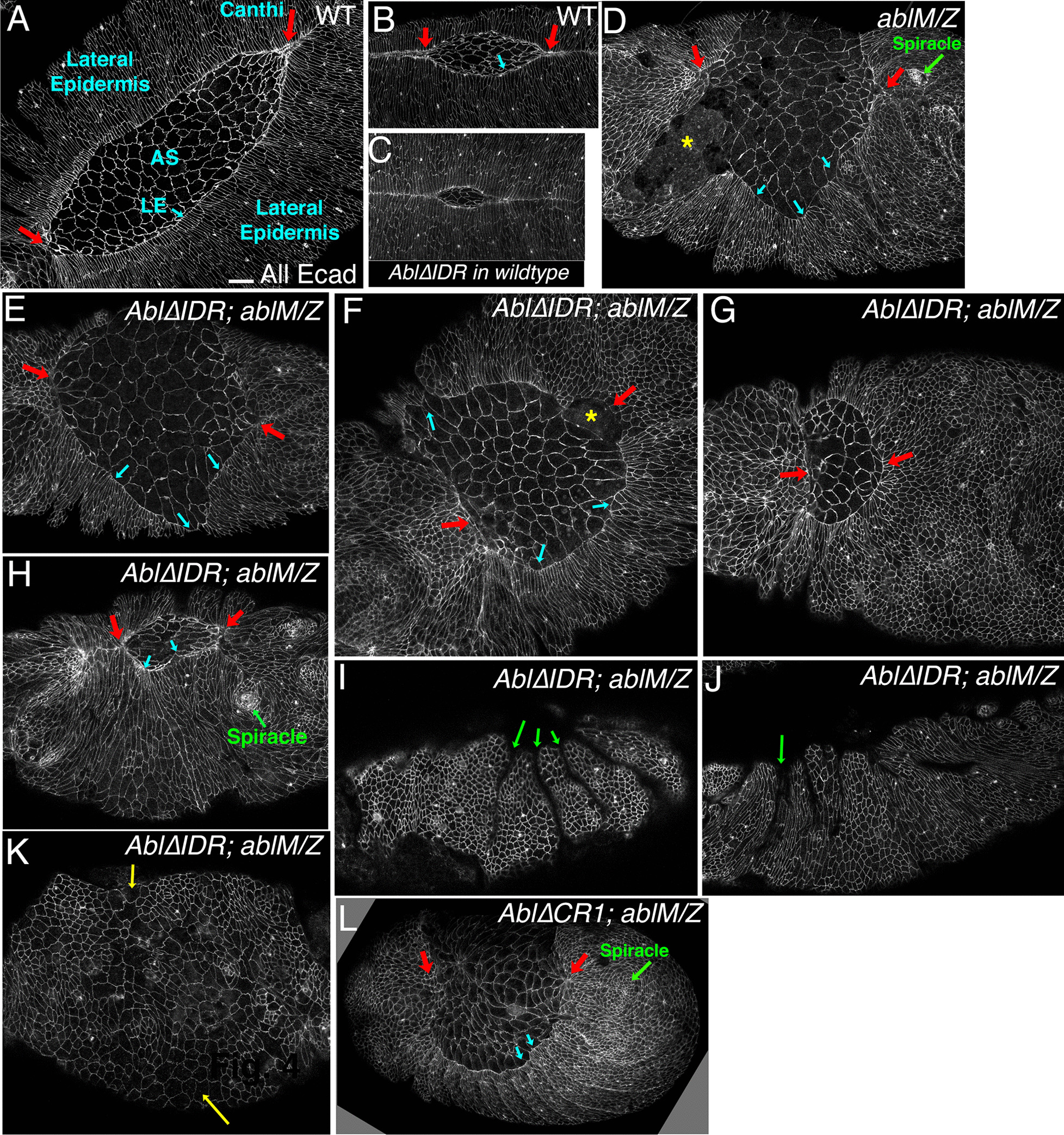
Abl∆IDR does not rescue defects in germband retraction or dorsal closure. Embryos stage 13–14, anterior left, dorsal (**a**–**g**) or lateral (**h**–**l**) views, stained with antibodies to Ecad to visualize cell shapes. **a**, **b** Wildtype embryos, dorsal view, at successively later stages of dorsal closure. The embryo is enclosed ventrally and laterally by epidermis but the dorsal surface remains covered by the amnioserosa (AS). The leading edge is straight (blue arrows) and as closure proceeds the epidermis meets and zips at the canthi (red arrows). **c** Embryo expressing Abl∆IDR in a wildtype background. Dorsal closure proceeded normally. **d** Representative *ablM/Z* mutant. Dorsal closure and germband retraction are disrupted. The spiracles remain dorsal (green arrow), the leading edge is wavy rather than straight (blue arrows), zipping at the canthi is slowed or halted (red arrows), and in places the amnioserosa has ripped from the leading edge, exposing underlying tissue (asterisk). **e**–**h**. *Abl∆IDR; ablM/Z* mutants, illustrating the range of defects in dorsal closure. **e**, **f** More typical *Abl∆IDR; ablM/Z* mutants, with a very wavy leading edge (blue arrows), slowed zippering at the canthi (red arrows), and ripping of the amnioserosa from the epidermis (asterisk)-22/33 embryos observed were in this category. **g** *Abl∆IDR; ablM/Z* mutant where zippering has happened at the posterior canthus but not the anterior one (red arrows). **h** Relatively mild phenotype, with closure nearly completed However, the leading edge is wavy (blue arrows) and the spiracles are present dorsally, revealing failure to complete germband retraction (5/33 embryos observed resembled these). **i**–**k** Most severe class of *Abl∆IDR; ablM/Z* mutants, in which the epidermis is reduced in extent, very deep and persistent segmental grooves remain (**i**, **j**, green arrows) and presumed multinucleate cells are often observed (**k** yellow arrows)—6/33 embryos observed resembled these. **l** *Abl∆CR1; ablM/Z* mutant for comparison. Scale bar = 15 µm

The morphogenetic events in which Abl’s roles have been analyzed in greatest detail are two of the final morphogenetic movements of embryogenesis: germband retraction and dorsal closure [31, 36]. These events are easily visualized by staining embryos with antibodies to E-cadherin (Ecad) to outline cells. At the end of stage 11 of wildtype embryogenesis, the caudal end of the embryo is curled up on the dorsal side. During stage 12, the germband retracts, ultimately positioning the tail end of the embryo at the posterior end of the egg, and thus leaving structures like the spiracles at the posterior end (Fig. 3a, arrow) and out of the dorsal view. At this stage, the ventral and lateral side of the embryo are enclosed in epidermis, but the dorsal side is covered by a “temporary” tissue, the amnioserosa (AS, Fig. 5a). During dorsal closure, the epidermis and the amnioserosa work in parallel to completely enclose the embryo in epidermis (reviewed in [52, 53]. Pulsatile apical constriction of the amnioserosal cells exerts force on the epidermis. In parallel, cells at the leading edge of the epidermis assemble a contractile actin cable, anchored cell–cell at leading edge tricellular junctions–this keeps the leading edge straight (LE, Fig. 5a, b blue arrows) and is important for zippering the epidermis together as the sheets meet at the canthi (Fig. 5a, b, red arrows). Actin-based protrusions from leading edge cells also aid in cell matching/alignment between the two sheets. *ablM/Z* mutants have defects in both germband retraction and dorsal closure [31, 36]. Germband retraction is not completed and the spiracles are thus positioned dorsally (Fig. 5d, green arrow). Dorsal closure proceeds very abnormally and often fails to go to completion. The leading edge is highly wavy rather than straight (Fig. 5d, blue arrows) and zippering at the two canthi is slowed (Fig. 5d, red arrows). Tissue tearing is often observed at the border between the leading edge and amnioserosa, leaving underlying tissue exposed (Fig. 5d, asterisk).

We thus asked whether these defects are rescued by Abl∆IDR. In wild type embryos that harbor the Abl∆IDR transgene, dorsal closure proceeds normally (Fig. 5c). However, *Abl∆IDR; ablM/Z* mutants have severe germband retraction and dorsal closure phenotypes reminiscent of what is seen in *ablM/Z* embryos. Occasional *Abl∆IDR; ablM/Z* embryos succeeded in proceeding through closure, but even these exhibited defects in germband retraction, with the spiracles positioned dorsally (Fig. 5h, green arrow), or problems with zippering at one of the canthi (Fig. 5g, red arrows; together these were observed in 5/33 embryos examined; Table 4). In most embryos closure was highly aberrant (22/33 late stage embryos examined; Table 4). The leading edge was wavy instead of straight (Fig. 5e, f vs. a, b, blue arrows). Zippering at the canthi was slowed (Fig. 5e, f red arrows) and often did not proceed uniformly, with zippering slower or absent at the anterior end (Fig. 5g, red arrows). As we observed in unrescued *ablM/Z* mutants, tearing occurred between the leading edge and the amnioserosa (Fig. 5f, asterisk). The proportions of the embryos in these two categories were roughly similar to those seen in unrescued *ablM/Z* embryos, in which 6/29 proceeded through closure while 22/29 had severe defects in the process (Table 4). This spectrum of defects were broadly similar to those we previously observed in *Abl∆CR1; ablM/Z* mutants (Fig. 5l; [36], suggesting this motif in the IDR is important for this function.

In a subset of Abl∆IDR; *ablM/Z* embryos (6/33 embryos examined; Table 4), the phenotypes at stages 13 and 14 were even more severe. These embryos had reduced epidermal coverage (Fig. 5i–k), suggesting earlier cell death. They also exhibited deep, un-retracted segmental grooves during dorsal closure (Fig. 5i, j green arrows), another known phenotype of *ablM/Z* mutants [36]. Some late stage *Abl∆IDR; ablM/Z* embryos retained very large cells (Fig. 5k, yellow arrows), which we suspect are the multinucleate cells known to arise during cellularization and gastrulation in *ablM/Z* mutants. This severe class of embryos likely represents the subset whose cuticles show substantial epithelial disruption (Fig. 3), and was more frequent than we observed in unrescued *ablM/Z* mutants, in which 1/29 embryos had similar phenotypes (Table 4), consistent with the increased frequency of disrupted cuticles in Abl∆IDR; *ablM/Z* embryos (Table 1). These were also more severe than the defects observed in *Abl∆CR1; ablM/Z* mutants. Together, these data reveal that Abl∆IDR completely fails to rescue defects in germband retraction or dorsal closure. Intriguingly, our previous analysis revealed that kinase activity and the FABD are largely dispensable for these morphogenetic events, while the CR1 PXXP motif within the IDR plays a role [36].

### Abl’s IDR is essential for its role in regulating leading edge cell shape and in actin regulation

**Fig. 6:**
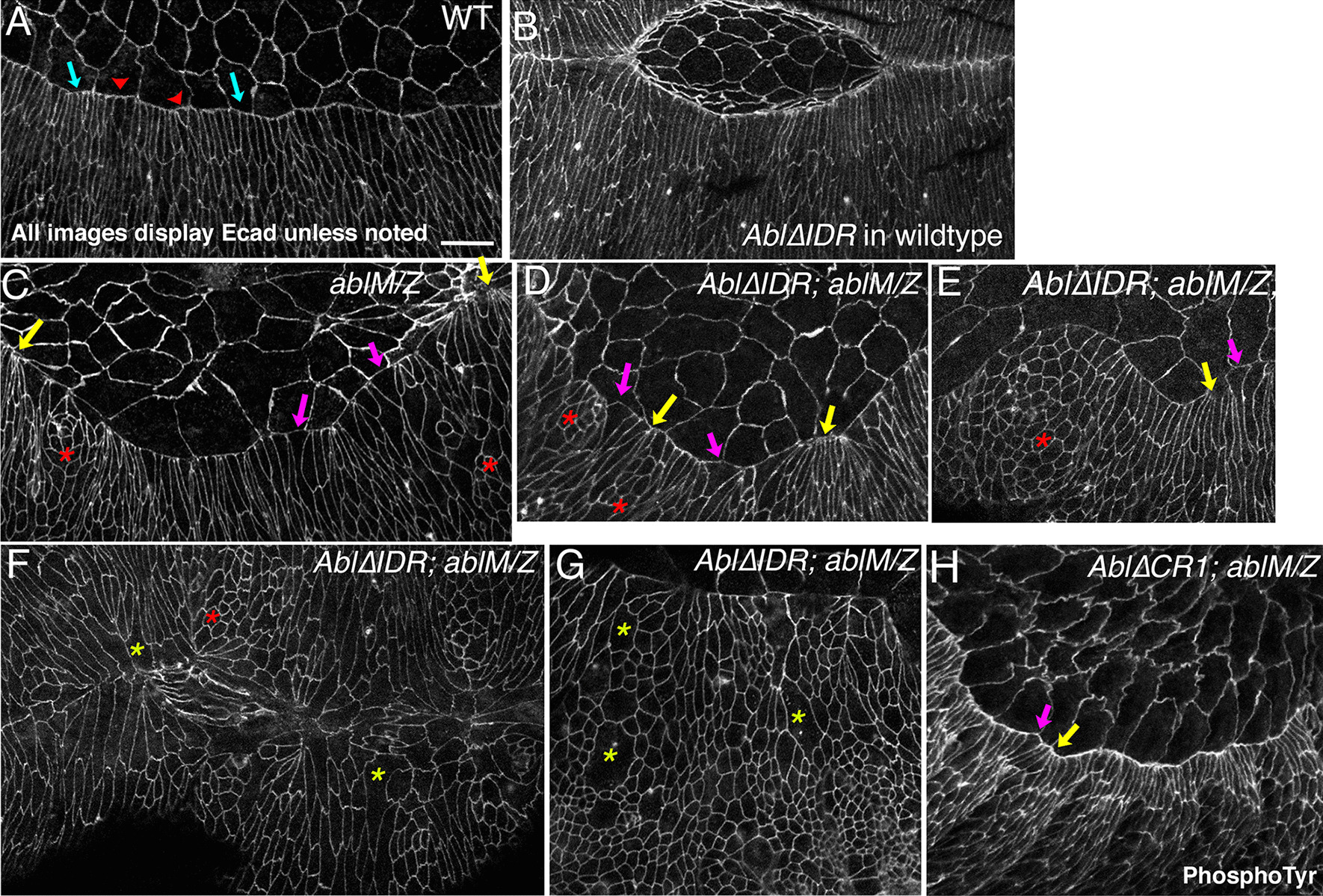
Abl∆IDR does not rescue defects leading edge cell shape. Leading edge, stage 13–14 embryos, anterior left, dorsal up unless noted, stained with antibodies to Ecad to visualize cell shapes. **a** Wildtype. The leading edge is straight, with even cell widths at the leading edge (blue arrows), excepting the slightly increased width at the positions of segmental grooves (red arrowheads). Scale bar = 10 µm. **b** Expressing Abl∆IDR in a wildtype background did not produce apparent defects in cell shape change during dorsal closure. **c** *ablM/Z* mutant, exhibiting the characteristic defects in leading edge cell shape. Leading edge cells are uneven in width, with some splayed open (magenta arrows) and some hyperconstricted (yellow arrows). Groups of cells also fail to elongate (red asterisks). **d**, **e** Representative *Abl∆IDR; ablM/Z* mutants. As in the unrescued mutant, leading edge cells are uneven in width, with some splayed open (magenta arrows) and some hyperconstricted (yellow arrows). Groups of cells also fail to elongate (red asterisks). **f** *Abl∆IDR; ablM/Z* mutant, dorsal view. Similar cell shape defects are seen in embryos that have completed or almost completed closure. **g** *Abl∆IDR; ablM/Z* mutant. Green asterisks indicate large, presumed multinucleate cells. **h** *Abl∆CR1; ablM/Z* mutant for comparison

We next explored the role of Abl’s IDR at the cellular and subcellular level. During dorsal closure the leading edge cells assemble a contractile actin cable that exerts tension along the dorsal cell margin. This cable maintains a straight leading edge, and together with amnioserosal apical constriction, elongates epidermal cells along the dorsal–ventral axis (reviewed in [52, 53]. The cable is anchored cell-to-cell at leading edge adherens junctions. In wildtype embryos tension along the cable is balanced among the cells and thus they exhibit relatively uniform shapes (Fig. 6a, arrows), with slight deviation at the segmental grooves (Fig. 6a, arrowheads). Expressing Abl∆IDR in a wildtype background caused no apparent defects in these cell shape changes (Fig. 6b). However, loss of Abl disrupts leading edge cell shapes, with some cells hyper-constricted and other splayed open (Fig. 6c, yellow and magenta arrows; 7.29 hyper-constricted or splayed open cells per leading edge vs. 0.68 in wildtype; Table 3; the full data set is in Additional file 4: Table S3), presumably due to failure of the leading edge actin cable in some cells. In addition, some cells fail to change shape (Fig. 6c, red asterisk; [31, 36]. These defects were rescued by our wildtype Abl transgene (Rogers et al*.* 2016; Table 3). We thus examined if Abl∆IDR rescued these cell shape defects. *Abl∆IDR; ablM/Z* embryos exhibited no rescue of defects in leading edge cell shape, with splayed open and hyperconstricted cells (Fig. 6d, e magenta vs. yellow arrows; 7.15 hyper-constricted or splayed open cells per leading edge; Table 3). We also observed groups of cells that failed to elongate (Fig. 6d, e red asterisks), as we had previously observed in *ablM/Z* mutants [31, 36]. Cell shape defects were even observed in the occasional embryos which managed to close dorsally (Fig. 6f). Finally, most embryos exhibited another *ablM/Z* mutant phenotype [31, 32]: large, presumptive multinucleate cells, which in some embryos were very frequent (Fig. 6g, yellow asterisks). From these data we conclude that Abl’s IDR is essential for regulating leading edge cell shape. We previously observed similar defects in *Abl∆CR1* mutants (Rogers et al. 2016; Fig. 6h), though that analysis suggested the effect of the CR1 deletion on splayed open and hyperconstricted cells shapes was slightly less severe than that of deletion of the full IDR.

**Table 3:** Counts of splayed out and hyperconstricted cells per leading edge during dorsal closure in AblΔIDR

Genotype	Mean # of cells splayed out per leading edge	Mean # of groups of hyperconstricted cells per leading edge	Number of leading edges scored	Number of embryos scored
*Wildtype*	0.68	0.00	19	10
*Abl WT; Abl M/Z*	1.94	0.25	16	10
*Abl M/Z*	7.29	1.71	31	27
*Abl*Δ*IDR; Abl M/Z*	7.15	1.54	26	21

Mean counts of splayed out and hyperconstricted cells per leading edge during dorsal closure in AblΔIDR. For raw data used to generate this table see Additional file 4: Table S3. Examples of cells that are scored as having a splayed out leading edge are indicated in Fig. 6 by magenta arrows. Examples of groups of cells that are scored as having a hyperconstricted leading edge are indicated in Fig. 6 by yellow arrows

**Table 4:** Comparison of late stage embryo phenotypes

Genotype	Phenotypic classes					
	Completed dorsal closure		Failure of dorsal closure		Epithelial integrity disrupted	
	n =	%	n =	%	n =	%
*Abl M/Z*	6	20.7	22	75.9	1	3.4
*Abl*Δ*IDR; Abl M/Z*	5	15.2	22	66.7	6	18.2

Number and proportion of late stage embryos of each genotype that exhibited the indicated phenotypes

**Fig. 7:**
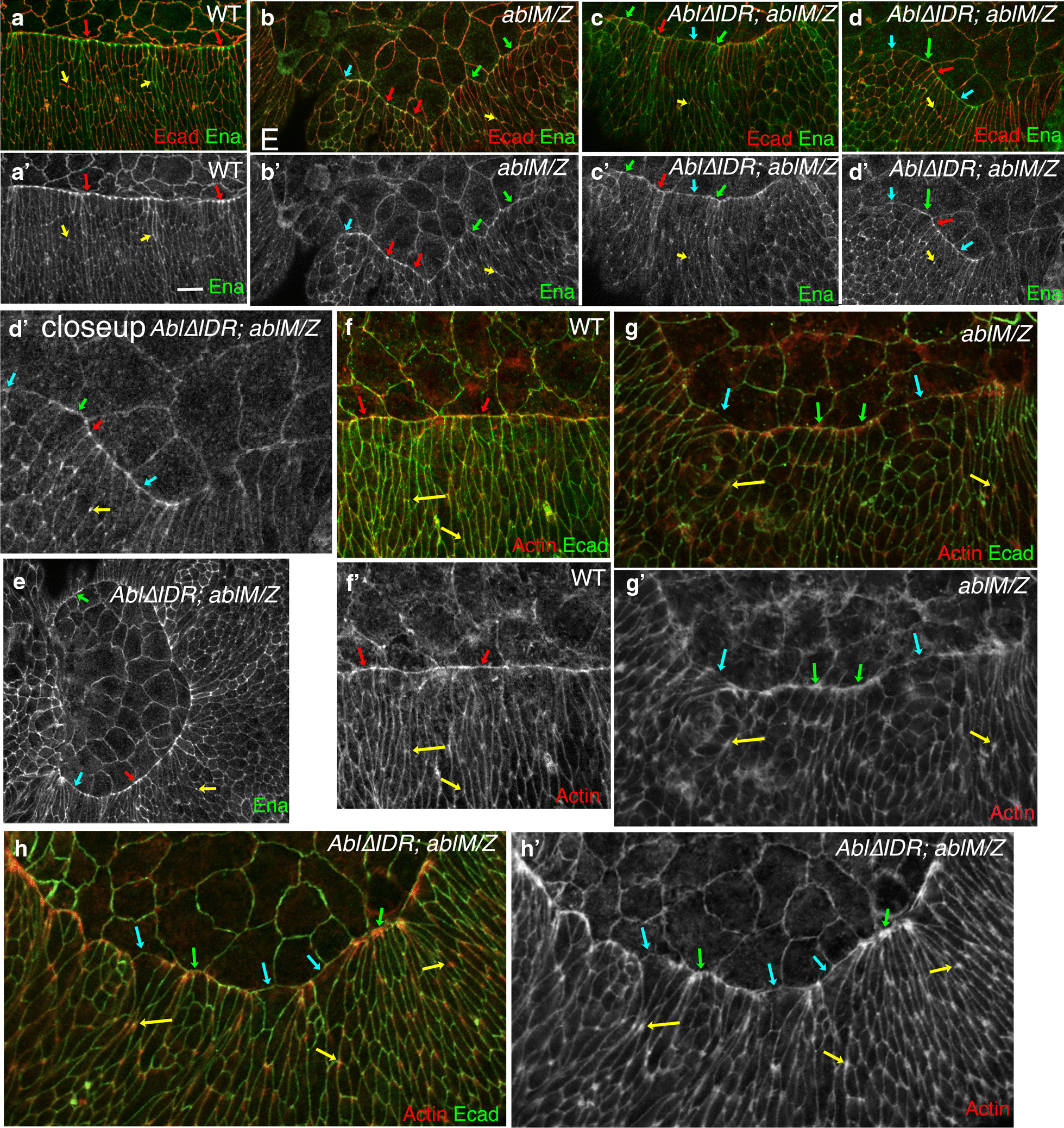
Abl∆IDR does not rescue defects in Ena localization or actin regulation. Leading edge, stage 13–14 embryos, anterior left, dorsal up, stained to visualize Ecad and Ena (**a**–**e**) or Ecad and F-actin (**f**–**h**). **a** Wildtype. Ena localizes cortically in both amnioserosal and epidermal cells. Ena is prominently enriched at leading edge tricellular junctions (red arrows), and is enriched at lower levels at tricellular junctions in the lateral epidermis (yellow arrows). Scale bar = 10 µm. **b** *ablM/Z* mutant. While Ena remains cortical and is enriched at lateral epidermal tricellular junctions (yellow arrows), uniform Ena enrichment at leading edge tricellular junctions is lost. While some tricellular junctions retain Ena enrichment (red arrows), at others Ena enrichment is reduced (cyan arrows) or Ena is found all along the leading edge (green arrows). **c**–**e** *Abl∆IDR; ablM/Z* mutants. No rescue is observed– Ena remains cortical and is enriched at lateral epidermal tricellular junctions (yellow arrows), but uniform Ena enrichment at leading edge tricellular junctions is lost. Some tricellular junctions retain Ena enrichment (red arrows), while at others Ena enrichment is reduced (cyan arrows) or Ena is found all along the leading edge (green arrows). **f** Wildtype. Actin is found cortically in all epidermal cells but is enriched in the leading edge actin cable (red arrows). **g** *ablM/Z* mutant. While most cells still have actin along the leading edge, actin intensity varies from lower (blue arrows) to much higher than normal (green arrows). Actin is also elevated at tricellular junctions of lateral epidermal cells (yellow arrows). **h** *Abl∆IDR; ablM/Z* mutant. Actin alterations are not rescued—while most cells still have actin along the leading edge, actin intensity varies from lower (blue arrows) to much higher than normal (green arrows), and Actin is elevated at many tricellular junctions of lateral epidermal cells (yellow arrows)

One of the key roles of Abl family kinases is regulation of the cytoskeleton. *Drosophila* Abl regulates the actin cytoskeleton through effectors like the actin polymerase Enabled (Ena). Our previous analysis suggests an important role for Abl regulation of Ena and actin at the leading edge during dorsal closure [31, 36, 54]. In wildtype embryos Ena localizes to the cell junctions of both amnioserosal and epidermal cells, but is strongly enriched in the tricellular junctions of leading edge cells, where the actin cable is anchored (Fig. 7a, red arrows; [54, 55]. Ena is also somewhat enriched at tricellular junctions of more ventral epidermal cells (Fig. 7a, yellow arrows). In *ablM/Z* mutants the uniform localization of Ena to leading edge tricellular junctions is lost (Fig. 7b; [36]. We thus asked whether Abl∆IDR can restore leading edge Ena localization. While Ena remained enriched at some leading edge tricellular junctions of Abl∆IDR; *ablM/Z* mutants (Fig. 7c–e red arrows), its uniform enrichment was lost, even though enrichment at lateral epidermal tricellular junctions remained (Fig. 7c– e yellow arrows). At many leading edge tricellular junctions Ena was weak or absent (Fig. 7c–e cyan arrows), and at other places Ena spread across the leading edge (Fig. 7c–e green arrows), all features we previously observed in *ablM/Z* mutants (Fig. 7b) and in embryos lacking the CR1 PXXP motif [36].

The altered cell shapes observed in *ablM/Z* mutants reflect defects in the leading edge actin cable [36]. In wildtype embryos the actin cable extends relatively uniformly across the leading edge (Fig. 7f, arrows), joined cell to cell at leading edge tricellular junctions. In contrast, in Abl∆IDR; *ablM/Z* embryos, the leading edge actin cable was discontinuous, with regions of reduced intensity (Fig. 7g, h, cyan arrows) interspersed with regions of elevated actin intensity (Fig. 7g, h, green arrows), as we previously observed in *ablM/Z* mutants [36]. Actin levels were also elevated at many lateral epidermal tricellular junctions (Fig. 7g, h yellow arrows) a featured shared by *ablM/Z* mutants [36] and by embryos in which Ena levels were artificially elevated [56]. These results indicate that Abl∆IDR does not rescue the defects in Ena localization or actin regulation seen after loss of Abl.

### The IDR is not essential for cortical enrichment of Abl protein

**Fig. 8:**
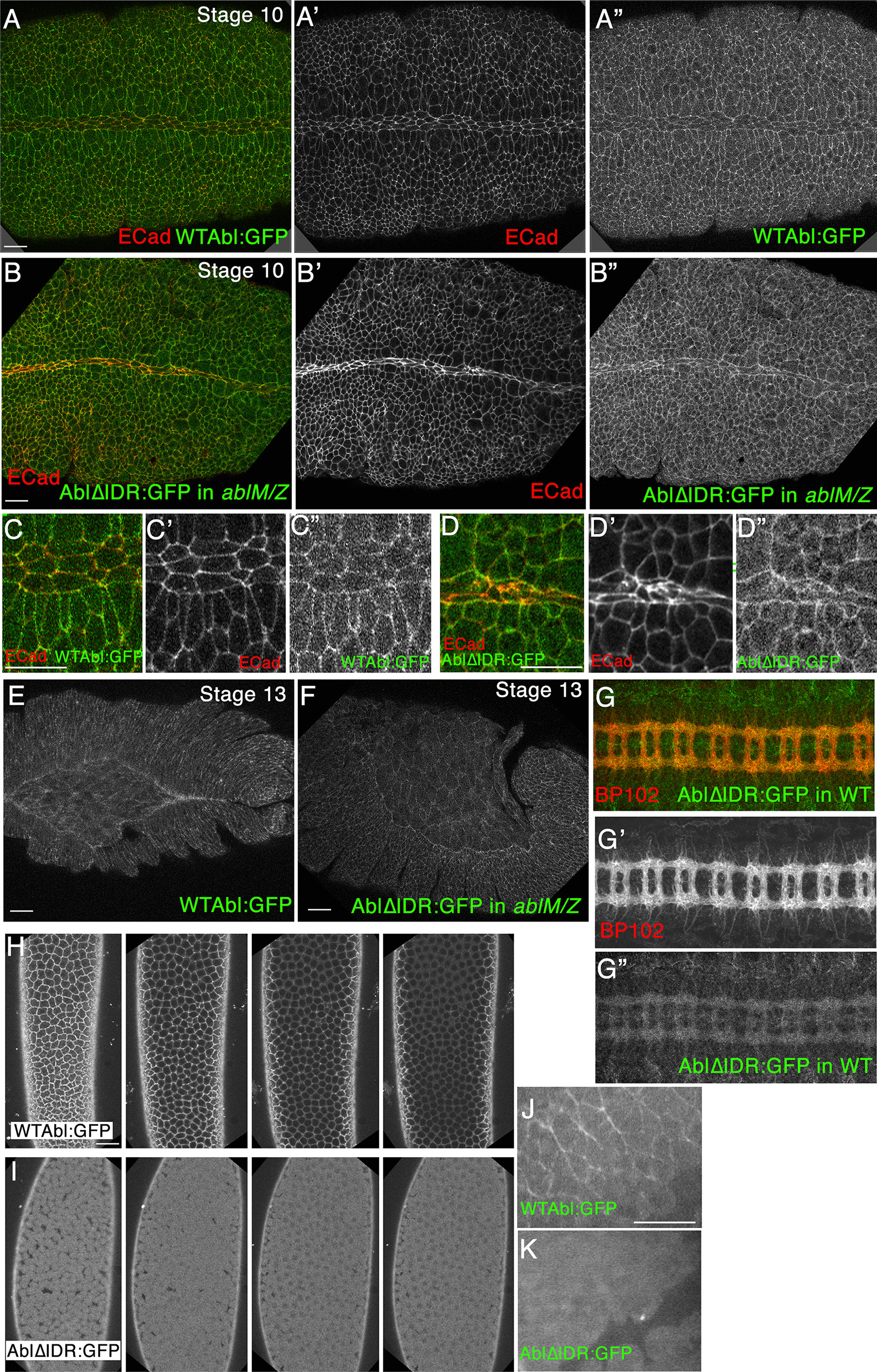
Abl∆IDR:GFP encodes a stable protein that remains enriched at the cell cortex, like wildtype Abl. **a**–**g** Embryos, stages indicated, anterior left. Fixed and stained for Ecad, with the GFP-tagged Abl proteins directly visualized by GFP fluorescence. Abl∆IDR:GFP was expressed in an *ablM/Z* mutant background except in **g**, where it was expressed in a wildtype background. Scale bars = 15 µm. **a**–**d**. During the extended germband stage, both wildtype Abl:GFP (**a**, **c**) and Abl∆IDR:GFP (**b**, **d**) have a cytoplasmic pool and are enriched at the cell cortex, as we previously observed is the case for endogenous Abl. **e**, **f** Cortical enrichment of both wildtype Abl:GFP and Abl∆IDR:GFP is reduced during dorsal closure. **g** When expressed in a wildtype background, Abl∆IDR:GFP is enriched in axons in the central nervous system, which are marked by staining of the BP102 antibody. **h**, **i** Live imaging of syncytial stage embryos, which were imaged at planes 1, 2, 3 and 4 µm below the vitelline membrane. Wildtype Abl:GFP (**h**) is clearly cortically enriched but, in contrast, Abl∆IDR:GFP (**i**) is found throughout the cell. **j**, **k** Live imaging of stage 8 embryos during germband extension

The data above illustrate how the IDR is critical for Abl’s mechanisms of action, as it is essential for most or all of Abl’s normal functions during morphogenesis. To further explore the importance of the IDR in Abl’s mechanism of action, we first examined the hypothesis that deletion of the IDR destabilized Abl protein or led to a change in its subcellular localization. In fixed embryos, wildtype Abl is found in a cytoplasmic pool and is enriched at the cell cortex. Cortical enrichment is strong in early embryos and gradually reduces through the end of dorsal closure [31–33]. Our previous analyses revealed that kinase activity and the FABD are dispensable for cortical localization, as are each of the four conserved motifs within the IDR [36]. To determine if there are redundant motifs in the IDR that lead to this result, we asked if Abl∆IDR retained the ability to localize to the cortex. We examined this in the background of *ablM/Z* mutants to eliminate the possibility of cortical recruitment via interaction with the wildtype Abl protein. At the extended germband stage endogenous Abl is enriched at the cortex, and this is mimicked by our wildtype Abl:GFP protein (Fig. 8a, c; [33, 36]. Abl∆IDR:GFP showed a similar degree of cortical enrichment at this stage (Fig. 8b, d). Cortical enrichment of both wildtype Abl:GFP and Abl∆IDR:GFP was diminished during dorsal closure (Fig. 8e, f). Intriguingly, when expressed in the wildtype background Abl∆IDR also retained the ability to be enriched in axons of the central nervous system (Fig. 8g), like wildtype Abl or wildtype Abl:GFP [36]. Thus Abl∆IDR encodes an apparently stable protein that retains the ability to associate with the cortex.

### Abl∆IDR protein is more stable than wildtype Abl protein

We next visualized the Abl:GFP and Abl∆IDR:GFP proteins live, without fixation. We first looked at syncytial stage embryos, sectioning down from the eggshell in 1 µm intervals. While cortical enrichment was obvious for Abl:GFP (Fig. 8h), it was much less apparent for Abl∆IDR:GFP (Fig. 8i)—instead, the entire cell appeared to be filled with protein. We also examined embryos after the onset of gastrulation, and saw a similar pattern—the cortical enrichment of wildtype Abl was not seen with Abl∆IDR (Fig. 8j vs. k). These data prompted us to examine a second hypothesis: deletion of the IDR led to elevation of accumulation level. All of our transgenes were driven by the endogenous *abl* promotor, which drives expression of transgenes at normal levels [33] and in our second set of transgenes we targeted all to the same chromosomal location to reduce the possibility of position effects. Immunoblotting had previously revealed that our wildtype GFP-tagged Abl and each of our previously analyzed mutants accumulate at levels similar to endogenous wildtype Abl [36]. We thus repeated this analysis with Abl∆IDR.

**Fig. 9:**
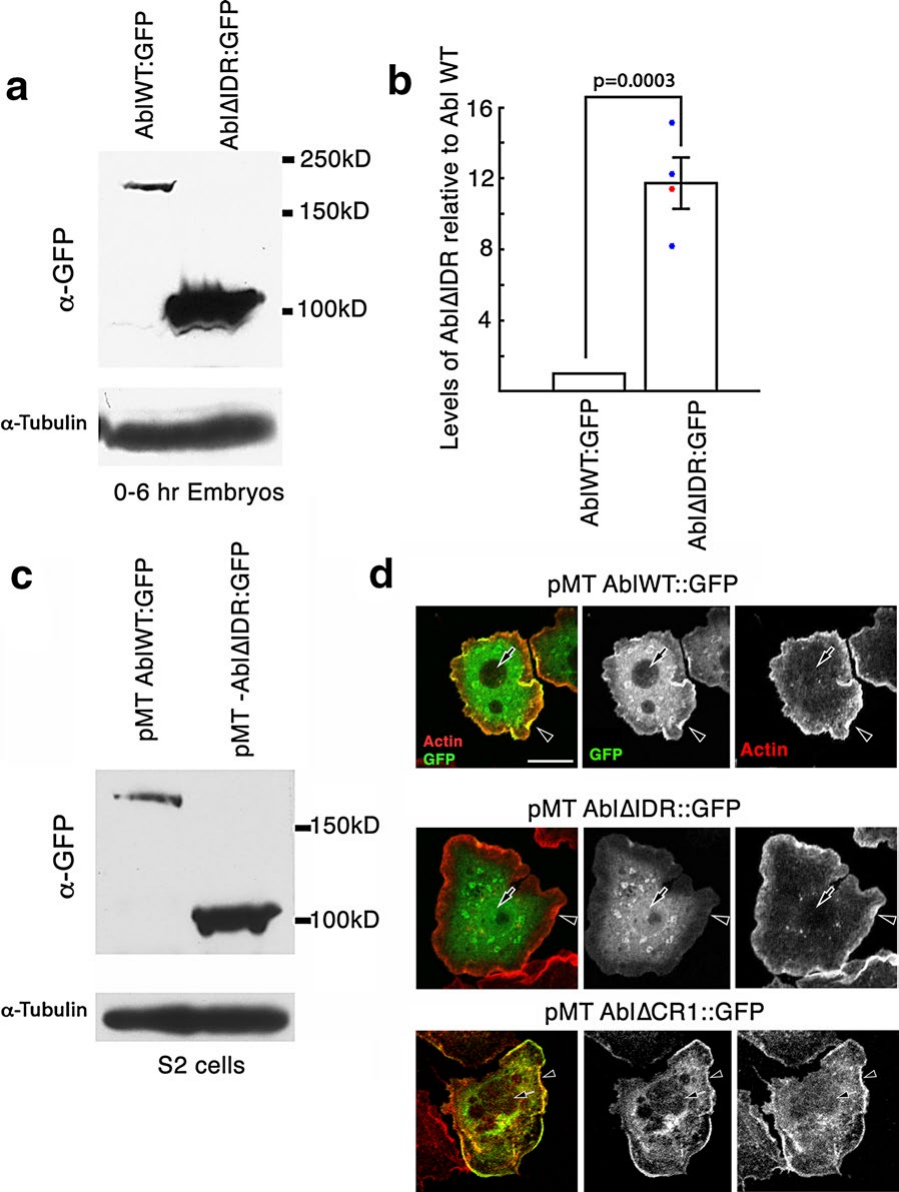
Abl∆IDR protein accumulates at much higher levels than wildtype Abl. **a** Immunoblot of 0–6 h embryonic extracts, blotted with antibody to GFP to detect our transgenic proteins. Tubulin serves as a loading control. Despite the fact that both transgenes are driven by the same endogenous *abl* promotor and the transgenes are at the same chromosomal location, Abl∆IDR protein accumulates at much higher levels than wildtype Abl. **b** Quantification of mean protein levels from four immunoblots, normalized to both wildtype Abl:GFP and using the loading controls. Colored dots indicate values of the individual blots (Values: 8.2, 11.4, 12.3, and 15.2, Mean: 11.7; Red dot indicates blot shown in **a**). Error bar = standard error of the mean. **c** Immunoblot of extracts of Drosophila S2 cells expressing transgenes encoding wildtype Abl:GFP or Abl∆IDR (at similar transfection efficiencies (see Methods)), both under control of the metallothionine promotor, blotted with antibody to GFP to detect our transgenic proteins. **d** Representative images of transfected S2 cells stained to visualize F-actin and our transgenic Abl proteins. Wildtype Abl:GFP is enriched in the lamellipodium (arrowhead; highlighted by F-actin) and excluded from nuclei (arrow), while Abl∆IDR:GFP is not enriched in the lamellipodium or excluded from nuclei. Cells expressing Abl∆CR1:GFP (bottom row) resemble those expressing Abl:GFP (enriched in the lamellipodium (arrowhead), excluded from nuclei (arrow)). Scale Bar = 10 µm

To our surprise, in embryos, AblΔIDR protein accumulates to substantially higher levels than does wildtype GFP-tagged Abl (Fig. 9a); quantitative immunoblots revealed that protein levels are elevated 11-fold (Fig. 9b). This cannot be attributed to chromosomal position effects, as we observed similar elevation in protein levels in flies carrying each of two independently generated Abl∆IDR transgenes (flies carrying the P-element -mediated transgenes generated for our initial experiments and the phiC targeted transgenes). Because this result was so surprising, we expressed our transgenic proteins in a well-characterized *Drosophila* cultured cell line, S2 cells, where they were driven by the heterologous metallothionein promotor. Strikingly, AblΔIDR protein also accumulated to a significantly higher level than wildtype Abl protein in transfected S2 cells (Fig. 9c). Together, these observations ruled out the possibility that the higher levels of AblΔIDR protein accumulation are solely due to differences in transcription: in the embryos, transcription of both wildtype and Abl∆IDR transgenes is driven by the same 2 kb upstream *abl* promoter region, while in S2 cells, transcription of transgenes encoding wildtype Abl or AblΔIDR was driven by the same metallothionein promoter and the plasmids encoding them had essentially identical transfection efficiencies (see Methods). Wild-type Abl:GFP is excluded from the nucleus (Fig. 9d top, arrow) and enriched in the lamellipodium (Fig. 9d top, arrowhead). In contrast, Abl∆IDR was not enriched in the S2 cell lamellipodium (Fig. 9d middle, arrowhead), and also was not excluded from nuclei (Fig. 9d middle, arrowhead). One possibility is that the elevated levels of Abl∆IDR saturated its normal binding sites in the lamellipodium and filled the cell. It also is possible that loss of the IDR impaired recruitment to the lamellipodium. The localization differences seen between wild-type Abl:GFP and Abl∆IDR are not seen in an Abl transgene lacking the PXXP motif (AblΔCR1::GFP, Fig. 9d, bottom).These data suggest that AblΔIDR protein is more stable and resistant to degradation than wildtype Abl, revealing that Abl’s IDR contains an element, outside of the PXXP motif, that is important for regulating Abl protein levels.

## Discussion

The important roles of Abl kinase in embryonic development, the nervous system, adult homeostasis and oncogenesis make understanding its molecular function essential for both basic scientists and clinicians. Abl is a complex multidomain protein and we and others have assessed the roles of its kinase activity and its many protein interaction domains, helping reveal their roles in Abl’s mechanism of action. Here we extended that work, exploring the roles of its intrinsically disordered region (IDR). This revealed that the IDR is essential for Abl’s mechanism of action at multiple steps in *Drosophila* morphogenesis. It also revealed a mechanistic role for the IDR in negatively regulating protein stability.

As one of the first protein kinases implicated in cancer, attention initially focused on Abl’s kinase activity. This clearly is critical for function of the Bcr-Abl fusion protein found in chronic myeloid and acute lymphocytic leukemia, and drugs targeting kinase activity revolutionized treatment of these diseases [2, 3]. However, studies of Abl’s normal roles in both Drosophila and in mammals suggest kinase activity, while important, is not essential, as Abl lacking kinase activity retained residual function in vivo [35, 36, 43]. In a similar fashion, the C-terminal F-actin binding domain (FABD) and other cytoskeletal interaction motifs serve important functions in some contexts, but are not essential for protein function in others [9, 36, 37, 43].

### Abl’s IDR plays a critical role in Abl’s diverse functions in morphogenesis

Abl’s IDR is an interesting but poorly understood feature of Abl. IDRs are found in diverse proteins and have attracted increasing interest. They are sites of protein regulation via posttranslational modifications, often contain embedded protein interaction motifs, and by mediating multivalent interactions can play a role in phase transitions leading to the assembly of biomolecular condensates [39–41]. They contain regions of low-complexity sequence that are not well conserved, which mediate relatively non-specific interactions. IDRs also can contain short conserved motifs that mediate specific protein interactions. This is the case in Abl. Our previous analysis focused on four predicted protein binding motifs within the IDR that are well conserved among different insects, which we refer to as CR1 to CR4 [36]. To our surprise, three of these, including a putative consensus binding site for the Abl effector Ena, are dispensable for rescuing viability and fertility [36]. However, the PXXP motif embedded in CR1 proved important for function—Abl∆CR1 mutants exhibited reduced adult and embryonic viability and had defects in most but not all aspects of Abl function during embryonic morphogenesis. Cheong and VanBerkum similarly found important functions for this motif in supporting adult viability and embryonic axon guidance [9]. However, the data from both groups reveal that Abl∆CR1 retains residual function. Cheong and VanBerkum extended this analysis by deleting larger regions of the IDR, singly and in combination. These data further support the idea that the PXXP motif is the only individually essential region of the IDR. However, their gain-of-function assays and analysis of effects on protein localization suggest that the region containing the Ena-binding motif also contributes to axon localization and subtly to function.

Here we cleanly deleted the IDR while leaving the FABD intact, allowing us to directly determine whether other regions of the IDR have additional functions. Our new data strongly support this hypothesis. In our assays of embryonic morphogenetic events in which Abl has a known role, Abl∆IDR failed to rescue mesoderm invagination and maintenance of epidermal integrity, whereas Abl∆CR1, lacking only the PXXP motif, retained full or substantial function (Fig. 11; [36]). Consistent with our data, Cheong and VanBerkum found that deleting the first quarter of the IDR had stronger effects than simply mutating the PXXP motif [9, 46]. In fact, loss of the IDR reduced Abl function more substantially than any of our other previous alterations, including simultaneously eliminating kinase activity and the FABD [36], revealing that the IDR plays a critical role in Abl function during morphogenesis. These data further suggest multiple regions within the IDR likely contribute collectively to provide full function (Fig. 11)—these may include additional less well conserved protein binding motifs, sites of posttranslational modification, or regions of simple sequence that mediate less specific interactions.

We observed disruption of epidermal integrity when we used the Abl∆IDR transgene to rescue the progeny of *abl*^*4*^*/Df* females, and expressing Abl∆IDR failed to rescue this phenotype of *ablM/Z* mutants. In fact, *Abl∆IDR; ablM/Z* mutants had an elevated frequency of embryos with severe disruption of epidermal integrity (Fig. 11). We suspect that the disruption of epidermal integrity we observe is a consequence of the early defects in syncytial development and cellularization, which in *ablM/Z* mutants is known to lead to the formation of multinucleate cells. Other mutants, including those that disrupt syncytial development and cellularization in different ways, as is seen in embryos mutant for the septin *peanut*, lead to a similarly disrupted cuticle phenotype [57]. Intriguingly, embryos maternally and zygotically mutant for the adapter protein Crk, which in mammals can bind the Abl PXXP motif and is thought to act as an Abl regulator or effector [11, 12], also have strong defects in syncytial development and cellularization, leading to strong disruption of epithelial integrity [45], as we observed here. Crk regulates actin dynamics in the early *Drosophila* embryo by recruiting SCAR to the cortex [45], and the PXXP motif within Abl’s IDR can bind proteins in the WAVE regulatory complex [46], of which Scar is a part. Together these data are consistent with an important role for the IDR in mediating Abl’s regulation of actin.

### Abl’s IDR plays a role in regulating Abl protein stability

Abl∆IDR protein has an additional property that casts light on the regulation of Abl: it accumulates at levels substantially higher than wildtype Abl. We observed this effect in *Drosophila* embryos with two different transgenes driven by the endogenous *abl* promotor inserted at different chromosomal locations, and, importantly, we also observed it when we expressed Abl∆IDR in cultured *Drosophila* cells driven by a heterologous promotor. These data imply that the IDR contains sequences that regulate Abl protein stability. None of our previous deletions of conserved motifs within the IDR (CR1-CR4) affected Abl levels [36], nor did the larger deletions of portions of the IDR made by Cheong and VanBerkum [9], suggesting this effect either involves a different region of the IDR or that it is a property of the IDR as a whole (Fig. 11).

**Fig. 10:**
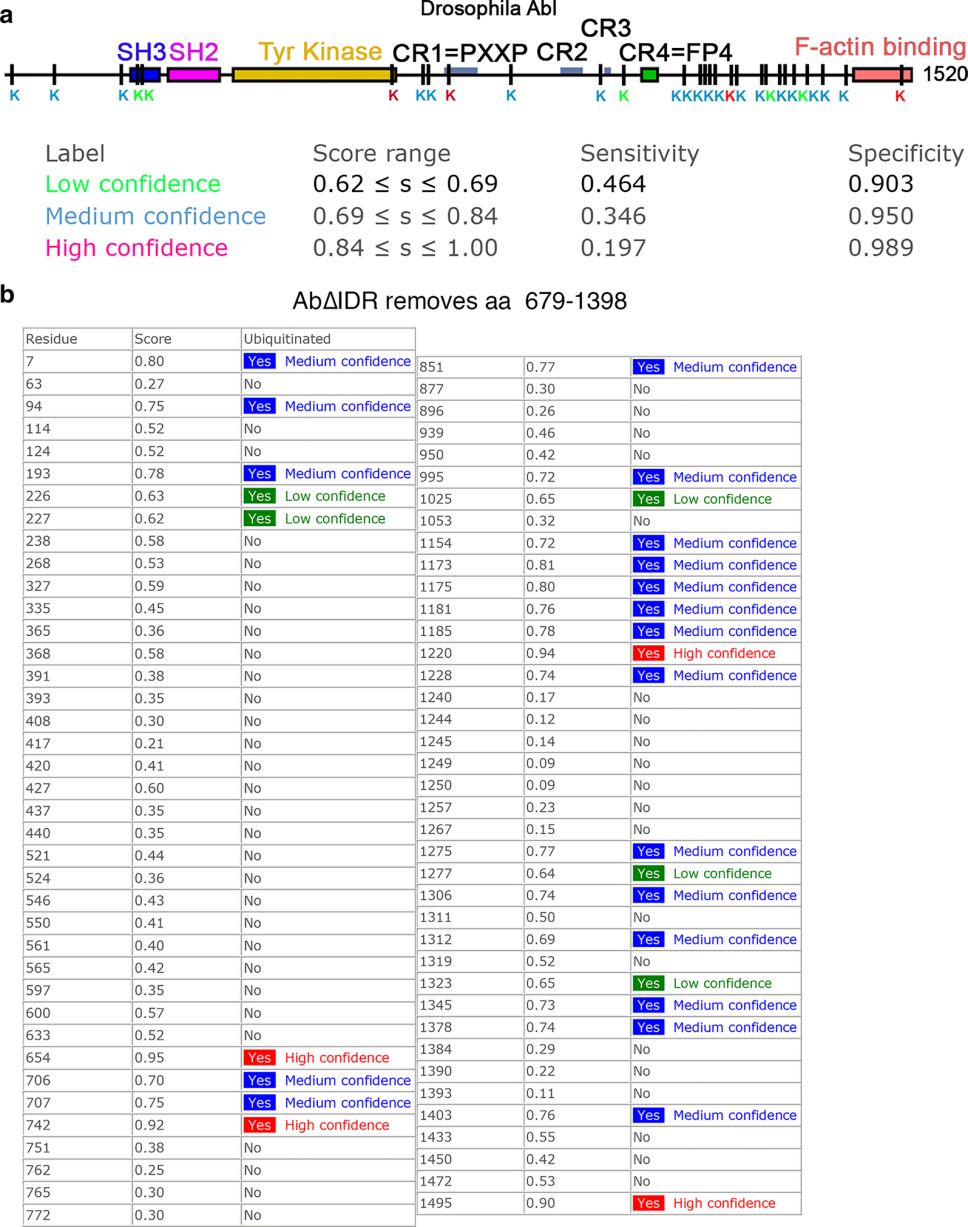
Many computationally predicted ubiquitination sites in Abl are in the IDR. Output of UbPred, computational prediction software to identify potential ubiquitination sites within Abl [60]. **a** Diagrammatic representation, showing high (red), medium (blue) and low (green) confidence predictions. **b** Table of amino acid positions of potential ubiquitination sites

IDRs have clearly defined roles in regulating protein stability, at least in part via their roles as preferred sites of posttranslational modifications. Almost 80% of known degrons reside in disordered regions [58], while computational predictions suggest a large fraction of ubiquitylation sites are in disordered regions [59, 60]. When we used the computational prediction software UbPred to identify potential ubiquitination sites within Abl [60], 19 of 24 medium and high confidence predicted ubiquitination sites were located within Abl’s IDR, and three more were in the disordered N-terminal region (Fig. 10a, b). The presence of an IDR in a protein also accelerates proteasomal degradation, and they can act as initiation sites for proteolysis [61]. Additionally, Ng et al. found that presence of IDRs may serve an important role in mediating ubiquitination in response to heat shock [62]. Abl’s long IDR also exceeds the length threshold observed for acting as an internal proteasome initiation site [63]. Taken together, this evidence strongly suggests that Abl’s IDR may play a role in ubiquitin-mediated protein turnover as a mechanism for Abl proteostasis. Mammalian Abl is regulated by the ubiquitin–proteasome system [64] and Abl can be ubiquitinated by the E3 ligase Cbl [65]. Mammalian Arg is also ubiquitinated in response to oxidative stress [66]. Future work will determine if this is a conserved property of the IDR across the Abl family, and by what mechanism this occurs. It will also be of interest to explore whether other regulatory post-translational modifications occur in the IDR.

One intriguing fact is that Drosophila *abl* encodes two alternative exons which are included or excluded in the multiple isoforms identified by the genome project. Both are located in predicted IDRs, an 18 amino acid exon in the region N-terminal to the SH3 domain and a 115 amino acid exon in the C-terminal part of the long IDR studied here. The isoform we used for our analysis, Abl-PG, includes the first alternative exon and excludes the second. Because Abl-PG fully rescues the null mutant, the second alternative exon doesn’t include any essential elements, but this does not rule out redundant roles for motifs and sequences here. It will be of interest to extend analyses and explore the role of these alternately spliced regions.

### Some remaining questions

**Fig. 11:**
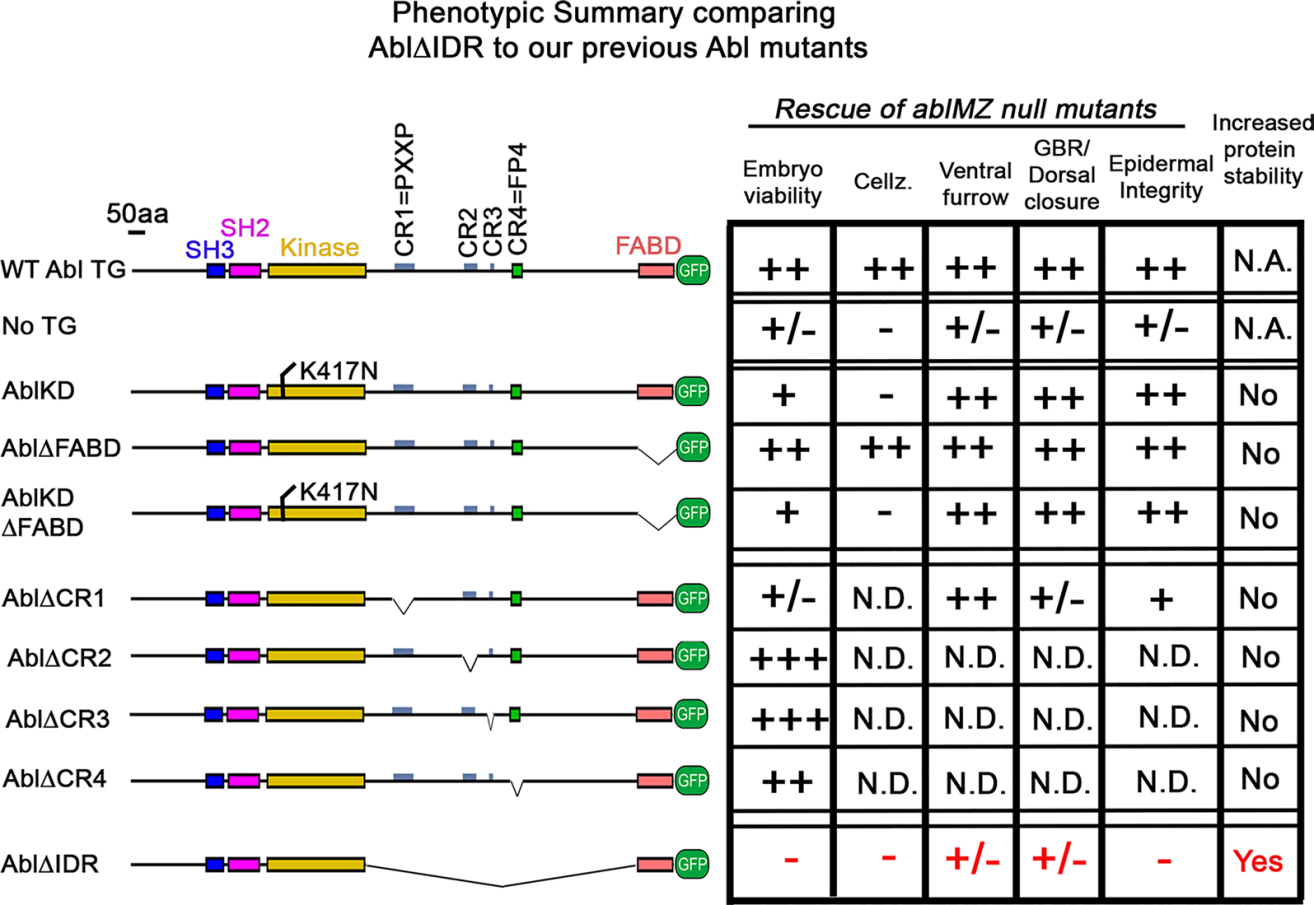
Phenotypic Summary comparing Abl∆IDR to our previous Abl mutants. N.D. = not done. N.A. = not applicable

In most cases, the phenotype of *Abl∆IDR; ablM/Z* mutants matches that of *ablM/Z* mutants, both in qualitative and quantitative terms. One remaining question is the mechanism by which expression of Abl∆IDR exacerbates the epidermal disruption phenotype of *ablM/Z* mutants (Fig. 11). This is not a classic dominant negative effect, in which a mutant interferes with the function of the wildtype protein. For instance, we saw no effect of Abl∆IDR expression in a wildtype background, while Abl∆IDR can worsen some phenotypes of the *abl*^*4*^ M/Z mutants, which lack wildtype protein. We also do not think this results simply from over-expressing Abl protein. In our earlier work we over-expressed wildtype Abl, leading to accumulation at levels similar to those seen here (up to ninefold). This led to only partially penetrant embryonic lethality, and dead embryos only had mild defects in head involution, without any effects on dorsal closure [67]. A second possibility is that Abl is part of multivalent protein complexes that retain some residual function in its absence, a property that would confer robustness. Abl∆IDR might be incorporated into these complexes and interfere with their activity. Phenomena like this have been variously referred to as type 2 second site non-complementation or “negative complementation,” and have been suggested to involve proteins that interact in multi-protein complexes. A third, though we think remote possibility is that the allele we use as an *abl* null allele [33], *abl*^*4*^, actually encodes a very low level of partially functional protein, via readthrough of the stop codon or a low level of downstream re-start. In this scenario, Abl∆IDR might interfere with the function of this residual Abl protein by forming inactive complexes with it or with some of its effectors or regulators. We think this is less likely as we could not detect Abl protein in *abl*^*4*^ mutants [33].

Another question involves the mechanism by which Abl regulates the cytoskeleton. Our own work and that of many others suggest that Ena is a major effector of Abl, and that Abl downregulates Ena activity. Consistent with this, reducing the dose of Ena can suppress the effects of Abl loss (e.g. [30, 32, 68]. Abl loss leads to re-localization of Ena to filopodial tips during dorsal closure [54], and elevated levels of apical Ena during cellularization [32], suggesting that Abl acts to restrict Ena localization or activity. However, the phenotypes we observe at the level of the whole embryo in *ablM/Z* mutants and in *Abl∆IDR; ablM/Z* mutants both have significant overlap with those observed in *enaM/Z* mutants [54]: all exhibit defects in germband retraction, dorsal closure (including difficulty in zippering together the epidermal sheets at the canthi), and leading edge cell shape. We suspect this reflects the fact that both Ena loss and Ena hyperactivity both disrupt actin regulation at the leading edge, likely in different ways at the molecular level, but that both disrupt the homeostasis required for balanced leading edge contractility. It’s also important to note that some phenotypes seen after Abl loss are not seen after Ena loss or overexpression (e.g., effects on cellularization or epithelial integrity, suggesting Abl regulates additional effectors. Defining how this happens in detail will require further work.

The mechanism by which Abl regulates Ena remains an open question. The genetic data clearly suggest that Abl negatively regulates Ena, but the molecular mechanisms remain unclear. In the syncytial and cellularizing embryo, Abl appears to prevent Ena localization to the apical region of the cells, as in its absence Ena is highly elevated there [32]. However, at the leading edge during dorsal closure, while loss of function of Abl does alter Ena localization, the effect is more complex, with loss of uniform enrichment at leading edge tricellular junctions, and reduction in overall Ena or spreading across the leading edge [36]. This may be a direct effect or may reflect alteration in where one would find actin plus ends. Resolving this mechanism is also an important future goal.

## Conclusions

In summary, our data provide new insights into the role of the intrinsically disordered region in an important signaling protein, the non-receptor kinase Abl. Abl’s key roles in normal development, tissue homeostasis and cancer have made it a subject of great interest, with analysis of its kinase activity and protein–protein interaction domains having attracted the greatest attention. Here we explored the function of a less well studied part of the protein, the long intrinsically disordered region between the kinase domain and the C-terminal actin binding domain. Our data reveal that it plays an essential role in embryonic morphogenesis, using Drosophila as a model. Abl’s regulation of cell shape change and the actin cytoskeleton all depend on the IDR—strikingly it is even more critical for protein function than is kinase activity (Fig. 11). Our data also reveal an unexpected role for the IDR in negative regulation of protein stability. These data will stimulate new explorations of the mechanisms by which the IDR regulates Abl stability and function, both in Drosophila and also in mammals. They also will stimulate further interest in the broader roles IDRs play in diverse signaling proteins.

## Supplementary Information

**Additional file 1: Figure S1.** Genetic crosses used to test adult viability or to examine maternal/zygotic mutants.

**Additional file 2. Table S1.** Scoring of cuticle phenotypes of abl mutants.

**Additional file 3. Table S2.** Counts of presumptive multinucleate cells in Ab∆IDR embryos.

**Additional file 4: Table S3.** Counts of splayed out and hyperconstricted cells per leading edge.

## Data Availability

*Drosophila* strains and plasmids are available upon request. The authors affirm that all data necessary for confirming the conclusions of the article are present within the article, Figures, Tables, and Supplemental Tables. Figure S1 show the crosses used for assessing embryonic and adult viability. Additional file 2: Table S1 has raw data for scoring mutant cuticles, Additional file 3: Table S2 contains raw data for the counts of multinucleate cells, Additional file 4: Table S3 contains raw data for the quantification of hyperconstricted and splayed out cell along the leading edge in dorsal closure.

## Abbreviations

Abi: Abl interacting protein
Abl: Abelson kinase
Arg: Abl-related gene
Arp: Actin-related protein
CR1: Conserved region 1
Crk: Chicken Tumor 10 Regulator of Kinases
Df: Deficiency
Ena: Enabled
FABD: F-actin-binding domain
GFP: Green fluorescent protein
IDR: Intrinsically disordered region
M/Z: Maternal/zygotic mutant
SCAR: Suppressor of cAMP receptors
SH2: Src homology 2
SH3: Src homology 3
WAVE: WASP-family verprolin-homologous protein
