# Supplementary figures and images for: Abelson kinase’s intrinsically disordered region plays essential roles in protein function and protein stability
Source: Cell Commun Signal. 2021 Feb 24;19:27. doi: 10.1186/s12964-020-00703-w (PMC7905622; doi:10.1186/s12964-020-00703-w)

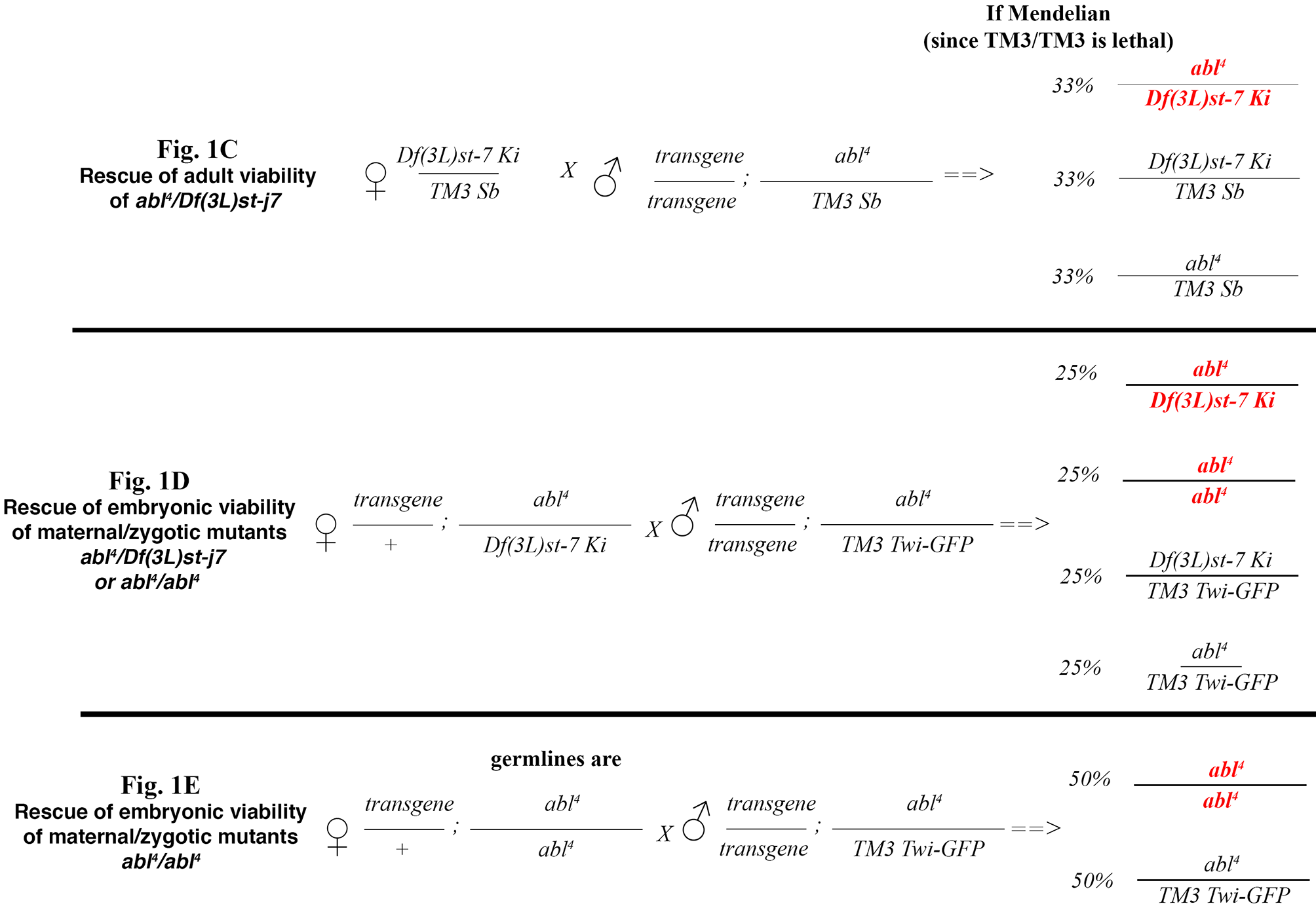

Supplement: Supplementary file 2 — Additional file 1: Figure S1. Genetic crosses used to test adult viability or to examine maternal/zygotic mutants. [file 12964_2020_703_MOESM2_ESM.tif]
